# Automated flow control of a multi-lane swimming chamber for small fishes indicates species-specific sensitivity to experimental protocols

**DOI:** 10.1093/conphys/coaa131

**Published:** 2021-01-07

**Authors:** Björn Illing, Andrea Severati, Justin Hochen, Paul Boyd, Paulin Raison, Rachel Mather, Adam T Downie, Jodie L Rummer, Frederieke J Kroon, Craig Humphrey

**Affiliations:** 1 ARC Centre of Excellence for Coral Reef Studies, James Cook University, 1 James Cook Drive, Townsville, Queensland 4811, Australia; 2 National Sea Simulator, Australian Institute of Marine Science, PMB 3, Townsville, Queensland 4810, Australia; 3 École Polytechnique Fédérale de Lausanne, School of Engineering, Route Cantonale, 1015 Lausanne, Switzerland; 4 College of Science and Engineering, James Cook University, 1 James Cook Drive, Townsville, Queensland 4811, Australia; 5 Australian Institute of Marine Science, PMB 3, Townsville, Queensland 4810, Australia; 6 Division of Research and Innovation, James Cook University, 1 James Cook Drive, Townsville, Queensland 4811, Australia

**Keywords:** Automation, CFD, fish larvae, particle tracking, swimming methodology

## Abstract

In fishes, swimming performance is considered an important metric to measure fitness, dispersal and migratory abilities. The swimming performance of individual larval fishes is often integrated into models to make inferences on how environmental parameters affect population-level dynamics (e.g. connectivity). However, little information exists regarding how experimental protocols affect the swimming performance of marine fish larvae. In addition, the technical setups used to measure larval fish swimming performance often lack automation and accurate control of water quality parameters and flow velocity. In this study, we automated the control of multi-lane swimming chambers for small fishes by developing an open-source algorithm. This automation allowed us to execute repeatable flow scenarios and reduce operator interference and inaccuracies in flow velocity typically associated with manual control. Furthermore, we made structural modifications to a prior design to reduce the areas of lower flow velocity. We then validated the flow dynamics of the new chambers using computational fluid dynamics and particle-tracking software. The algorithm provided an accurate alignment between the set and measured flow velocities and we used it to test whether faster critical swimming speed (*U*_crit_) protocols (i.e. shorter time intervals and higher velocity increments) would increase *U*_crit_ of early life stages of two tropical fish species [4–10-mm standard length (SL)]. The *U*_crit_ of barramundi (*Lates calcarifer*) and cinnamon anemonefish (*Amphiprion melanopus*) increased linearly with fish length, but in cinnamon anemonefish, *U*_crit_ started to decrease upon metamorphosis. Swimming protocols using longer time intervals (more than 2.5 times increase) negatively affected *U*_crit_ in cinnamon anemonefish but not in barramundi. These species-specific differences in swimming performance highlight the importance of testing suitable *U*_crit_ protocols prior to experimentation. The automated control of flow velocity will create more accurate and repeatable data on swimming performance of larval fishes. Integrating refined measurements into individual-based models will support future research on the effects of environmental change.

## Teaser

Swimming performance is an important metric for estimating locomotory capacity in fishes. Yet, most swimming chambers require manual control. We therefore developed an open-source algorithm to automate swimming chambers and control water velocity. Testing the critical swimming speeds of tropical fish offspring with different protocols revealed species-specific sensitivities to experimental protocols.

## Introduction

Knowledge on how aquatic animals perform under changing or altered environmental conditions is important not only for understanding the mechanistic basis for cause-and-effect responses but also for developing sustainable and ecosystem-based management strategies (Ward *et al.*, 2016; Álvarez-Romero *et al.*, 2018). For example, conservation and fisheries management benefit from growth, survival and dispersal models for early life stages of fishes to better predict and understand trends in fish population dynamics. These individual-based models can provide a valuable insight into how environmental factors scale from individual-level changes in performance to population-level trends, such as changes in the number of young fish being added to a population (i.e. recruitment) or overall offspring dispersal (i.e. population connectivity) (Hufnagl and Peck, 2011; Kendall *et al.*, 2016; Ward *et al.*, 2016; Faillettaz *et al.*, 2018; Bode *et al.*, 2019). However, many individual-based biophysical models lack sufficient biological data, such as information on swimming performance (Peck and Hufnagl, 2012). Thus, there exists a strong need for accurate, empirical data and highly controllable and reproducible experimental designs to investigate the swimming performance of early life stages of fishes.

Several methods have been developed to test the swimming performance of fishes, each tailored to answer specific physiological, behavioural and/or ecological questions. For example, measuring burst swimming and escape responses can aid in understanding predator–prey relationships (e.g. Allan *et al.*, 2017), whereas undisturbed *in situ* or tank observations provide information regarding routine swimming speeds, such as those at which fishes migrate and/or search for prey (e.g. Leis *et al.*, 1996; Fisher and Bellwood, 2003). Swimming chambers, where flow velocities can be manipulated, have been used to swim fishes to exhaustion, either at set speeds (endurance swimming; Majoris *et al.*, 2019) or by a step-wise increase in flow velocity (critical swimming speed, *U*_crit_) (Brett, 1964; see review by Ellerby and Herskin, 2013). Swimming methodologies can also differ based on the equipment or experimental protocol used, and some studies have systematically investigated the effects of step heights (velocity increments) and step lengths (time intervals) on critical swimming speeds in temperate fishes. In two studies on juvenile largemouth bass (*Micropterus salmoides*) and juvenile shortnose sturgeon (*Acipenser brevirostrum*), the authors found that slower protocols (i.e. lower velocity increments and longer time intervals) reduced the estimates of *U*_crit_ (Farlinger and Beamish, 1977; Downie and Kieffer, 2017). This information has supported the use of swimming performance as a proxy for whole-organism performance and condition in fishes, including their early life stages, and has led to management recommendations (e.g. for fishway use in rivers; Braaten *et al.*, 2015; Downie *et al.*, 2020). Surprisingly, and to our best knowledge, no studies have tested the effects of using different critical swimming performance protocols in marine fish larvae yet.

A popular experimental design used to test the swimming performance of larval fishes, a multi-lane swimming chamber, was originally described by Stobutzki and Bellwood (1997). Several studies have since used this design to investigate either species- and/or taxon-specific differences (Fisher *et al.*, 2005) or to describe the ontogenetic development of swimming performance in fishes (Clark *et al.*, 2005; Leis *et al.*, 2007; Faria *et al.*, 2009; Kashef *et al.*, 2014; Kopf *et al.*, 2014; Silva *et al.*, 2014). Moreover, the multi-lane swimming chamber design has been used to investigate the effects of environmental stress, such as the effects of match–mismatch scenarios with prey on swimming performance (Leis and Clark, 2005; Faria *et al.*, 2011a,b). In other studies, researchers have investigated the effects of elevated temperature (Moyano *et al.*, 2016), increased partial pressure of carbon dioxide (*p*CO_2_) (Munday *et al.*, 2009; Bignami *et al.*, 2013, 2014; Silva *et al.*, 2016) or both in combination (Watson *et al.*, 2018; Cominassi *et al.*, 2019). The original multi-lane swimming chamber was designed for high throughput of experimental animals and was a quick and cost-efficient assembly. Yet, the fluid dynamics of the setup were never examined or validated and the manual control of the system required constant operator presence.

With advanced technological innovations, several design and control optimizations have become available for swimming chambers. Applications, such as computer-aided design (CAD) and computational fluid dynamics (CFD) software, can help to develop and test swimming chambers and their flow dynamics virtually (Vezza *et al.*, 2020). Similarly, computer-controlled cutting or 3D printing machines assist with creating pieces for swimming flumes, often on very small scales (Huang *et al.*, 2020). At the same time, microcontrollers can control flow settings in swimming flumes more accurately, as they reduce delays in flow changes associated with manually operated valves (Widrick *et al.*, 2018). These advances can improve the accuracy and repeatability of measurements, which is particularly relevant when assessing multiple, combined environmental stressors on fish swimming performance. Furthermore, these optimizations could assist in tackling current reproducibility issues in ecology and evolution studies (Fraser *et al.*, 2018).

In this study, we aimed to automate multi-lane swimming chambers to improve the accuracy and repeatability of swimming performance measurements of small fishes. We therefore developed an algorithm to automate the flow control and to execute repeatable, complex flow patterns without delays in flow velocity. Furthermore, we made structural modifications to the original multi-lane swimming chamber (Stobutzki and Bellwood, 1997) and validated the flow dynamics using CFD and particle-tracking software. We then used the multi-lane swimming chambers and automated control to systematically investigate the effects of different test protocols on critical swimming speeds of two tropical fish species. More specifically, we hypothesized that faster protocols would increase the estimates of critical swimming speeds during early ontogeny in cinnamon anemonefish (*Amphiprion melanopus*) and barramundi (*Lates calcarifer*).

## Materials and methods

### Swimming chamber design

**Figure 1:**
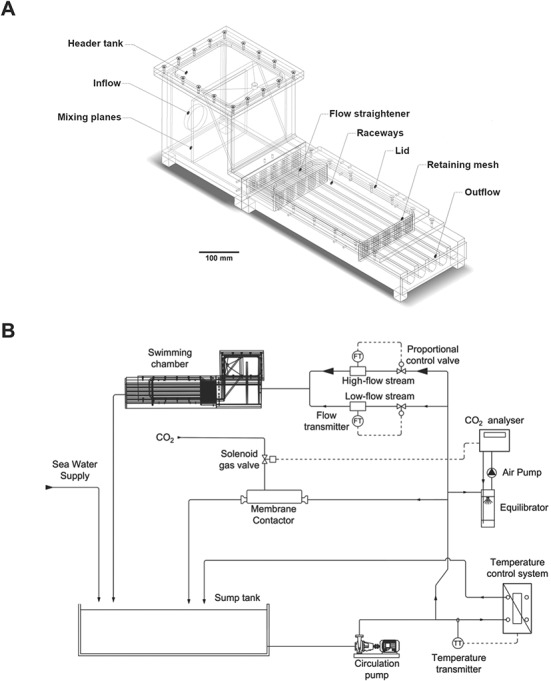
(**A**) Multi-lane swimming chamber, modified after Stobutzki and Bellwood (1997). Design modifications included rounded instead of rectangular-shaped raceways (244.0-mm length × 20.9-mm width ×37.3-mm depth) and flow straighteners that were doubled in length (80.0 instead of 40.0 mm). These changes helped (i) reduce the areas of lower flow velocity and (ii) increased flow uniformity. The drawing was created in CAD software and can be downloaded from the Supplementary Material S1. (**B**) Organizational chart of the SCADA system monitoring and regulating water parameters and flow velocity in the swimming chamber. PLC allows temperature and carbon dioxide partial pressure (*p*CO_2_) to be simultaneously checked and adjusted. Water flow can be controlled by two proportional valves (a high- and a low-flow one) that are adjusted according to the set flow velocity.

The design of the multi-lane swimming chamber was drafted using CAD software (AutoCAD Inventor®, Autodesk Inc, San Rafael, CA, USA), and the individual components were machined from polymethylmethacrylate panels using a computerized numerical control cutting machine (Mazak Integrex J-200, Yamazaki Mazak, Oguchi, Japan). The design was based on previously built, multi-lane swimming chambers (e.g. Stobutzki and Bellwood, 1997; Faria *et al.*, 2009) with modifications to the raceways, the flow-straightener and the flow control (Fig. 1A). The re-designed, multi-lane swimming chamber can be operated in a recirculating system to accurately control the flow as well as the water quality parameters, such as temperature and/or *p*CO_2_. Modifications of the raceways (244.0-mm length) comprised (i) rounded (max. height, 37.3 mm; max. width, 20.9 mm; cross-sectional area, 660 mm^2^) instead of rectangular bottoms to reduce areas of lower flow velocity and improve flow homogeneity (Aminian *et al.*, 2015) and (ii) doubling the length of the flow-straightener (80.0-mm length, 5-mm diameter perforations) to improve flow uniformity and minimize turbulence in the initial segment of the swimming lanes. Further, a perforated panel (5-mm diameter perforations) was added to the rear end of the swimming raceways to keep a finer plastic mesh in place that retained fatigued fish.

In our setup, the recirculation system is powered by a magnetic drive centrifugal pump (Iwaki magnet drive pump MX-400, Iwaki Co., Ltd, Tokyo, Japan). The water flow, delivered by the recirculating loop to the chamber, is monitored using flow transmitters (Type 8032 Bürkert, Ingelfingen, Germany) and regulated using proportional electric actuators (ER20.X3B.GP6, Valpes, Moirans, France) connected to two customized ball valves (DN15 and DN50 VKD series, FIP SpA, Italy). The main feature of the flow control loop (Fig. 1B) is regulating the two parallel streams of different size and capacity. The two streams, with 15-mm (low flow) and 50-mm (high flow) nominal diameters, respectively, are necessary to both measure and control flow with the required accuracy and repeatability over a wide range of experimental velocities. The incoming water first flows through the pre-chamber where the flow pattern is disrupted by two mixing planes before entering the flow straightener at the front end of the raceways. For our purposes, we built three multi-lane swimming chambers; all of which could be controlled independently. The Inventor® model is available in Supplementary Material S1.

### Automated flow control

**Figure 2:**
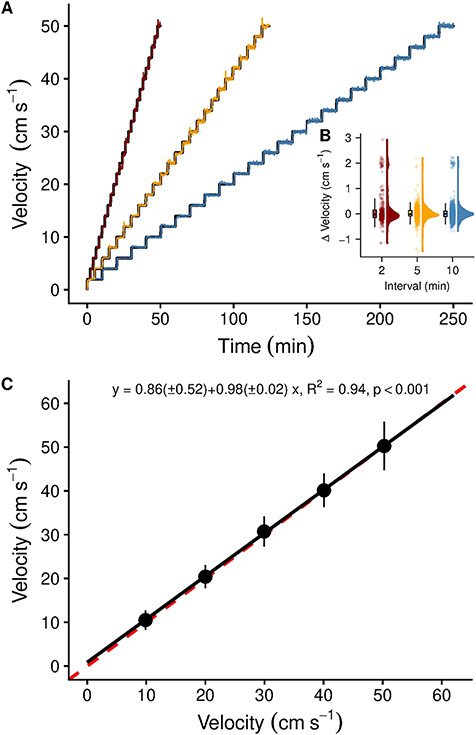
(**A**) Alignment of the automated set and measured flow velocities for critical swimming performance (*U*_crit_) protocols using 2-cm s^−1^ increments (step height) and three intervals (2, 5 and 10 min, step lengths); (**B**) summary statistics (outliers not shown), raw data and probability density of the difference between set and measured flow velocities across the three time intervals (colour-coded); (**C**) velocity validation of the swimming chamber using video recordings of neutrally buoyant, fluorescent green microspheres. All five raceways showed similar velocities and were not different from the ideal 1:1 line (red, dashed line). Flow visualization patterns were uniform and straight (see https://doi.org/10.5061/dryad.z34tmpgb7 for examples). Symbols and error bars represent mean and standard deviation (*n* = 50 per velocity increment).

The flow control was managed through a programmable logic controller (PLC, Siemens SIMATIC S7-1500) using an algorithm developed in-house (for TIA Portal, Siemens, Munich, Germany; see Supplementary Material S2) to allow for fully automated, accurate and repeatable adjustments of water velocity (Fig. 1B). The software algorithm integrates the feedback from the flowmeters and the valve opening to align the set value (SV) and the measured value (process value) for the flow velocity required. Any complex and/or iterative flow pattern can be programmed and executed reliably without further operator intervention and disruption to the behavioural and performance responses of the fish. Importantly, the swimming routine is remotely initiated and does not require the operator to be present in the experimental room. After several calibration trials, the software algorithm can accurately adjust the proportional valves to match the flow velocity SV for the full range of the swimming trial at the scheduled timeframe. We tested several standard ramping protocols to test the alignment of the set and measured flow velocities (Fig. 2A and B).

### Manipulation of environmental parameters

The multi-lane swimming chambers presented here have been designed to enable researchers to test the swimming performance of fishes under combined environmental stressors. Although environmental parameters were not manipulated in this study, temperature and *p*CO_2_ control was implemented in the swimming systems via the PLC system. This allows testing swimming performance under combined predicted ocean warming and acidification conditions in future projects.

In each of the swimming systems, *p*CO_2_ is measured continuously by an equilibrator system (as per Dickson*et al.*, 2007). Briefly, a side stream of water from the main circulation pump is provided in a fine spray to an air-tight chamber (equilibrator) and the *p*CO_2_ in the air within the head space of the chamber reaches equilibrium with the *p*CO_2_ of the experimental sea water. The air mixture is passed through the non-dispersive infrared CO_2_ analyser to measure *p*CO_2_ (Telaire T6613, Amphenol, PA, USA). The reading provides continuous feedback to the control system to dissolve aliquots of CO_2_ into the system and match the SV for *p*CO_2_; the gas dosing is controlled by the actuation of solenoid valves to deliver CO_2_ to a membrane contactor (3M™ Liqui-Cel™ EXF Extraflow 2.5X8) installed on a side stream line that returns CO_2_-enriched water to the system’s sump for homogenous mixing. The *p*CO_2_ values are logged at 20-second intervals.

The experimental temperature is also controlled by the PLC system via automatic valves and a shell and tube titanium heat exchanger (Waterco Heat Exchanger 56′). The feedback is provided to the control system by a temperature sensor (TC Direct, FEP Insulated RTD Pt100 Sensors coupled with a Pt100 4-20mATransmitter) positioned at the intake of the pump. The logic switches from cooling to heating mode and actuates the proportional control valve to vary the amount of primary water, 15°C and 40°C, which is delivered to the heat exchanger.

### Flow dynamics

#### Particle tracking

For flow visualization, we tracked neutrally buoyant particles in the newly designed swimming chambers. We used fluorescent green polyethylene microspheres (size range 850–1000 μm) treated with a biocompatible surfactant to achieve neutral buoyancy (Tween 20; both Cospheric LLC, Santa Barbara, CA, USA). The microspheres were recorded from above (UI-3180CP Rev. 2.1, IDS GmbH, Obersulm, Germany) under UV light at velocities ranging from 10 to 50 cm s^−1^ at 10-cm s^−1^ increments. The videos were processed in ImageJ (Rasband, 1997) using the Flowtrace algorithm for visualizing time-varying flow fields (Gilpin *et al.*, 2017), allowing us to analyse the distance the microspheres travelled per second (*n* = 10 per raceway and velocity increment). Unfortunately, we had no physical copy of the original swimming chamber with rectangular raceways available to compare particle-tracking results across designs.

#### Modelling

**Figure 3:**
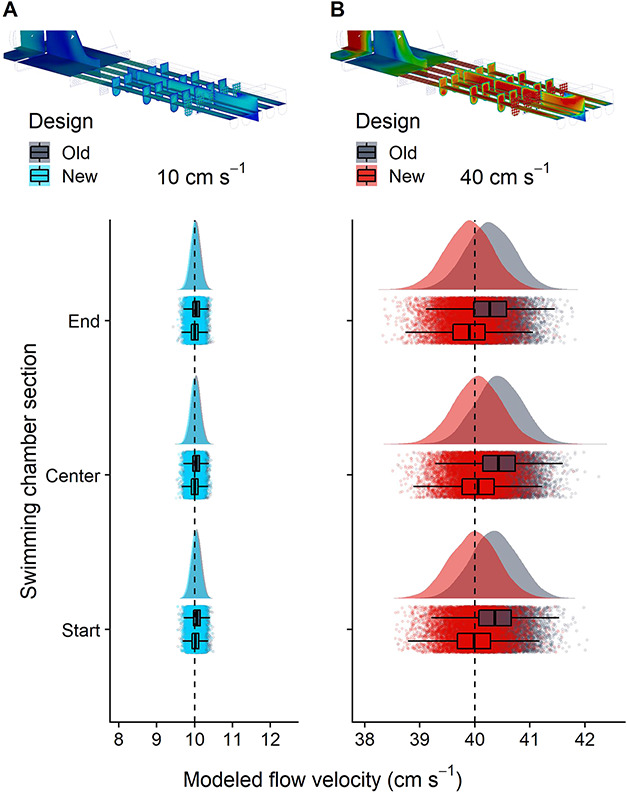
Modelled flow velocities across different swimming chamber sections and designs at (**A**) 10 cm s^−1^ and (**B**) 40 cm s^−1^. To improve flow uniformity and reduce areas of lower velocity, the new swimming chamber design had rounded instead of rectangular raceway bottoms and an elongated flow straightener. Using CFD software, flow velocities were modelled on grids at three sections of the swimming chamber (start, centre, end). Bayesian generalized linear models showed higher accuracy of the new swimming chamber design at the faster velocity but no overall differences in velocity across sections or designs. Data were simulated from the posterior predictive distributions of the Markov chain Monte Carlo models. Presented are the summary statistics (outliers not shown), the medians from each iteration and the probability density curves. Dashed lines indicate set flow velocities.

We used CFD software to model the flow dynamics in the original (old) and modified (new) swimming chambers (Autodesk® CFD, Autodesk Inc, San Rafael, CA, USA). We modelled flow velocities of 10 and 40 cm s^−1^, representing ecologically meaningful transport speeds that early life stages of tropical fishes can experience (see references in Fisher, 2005). In contrast to the previous designs (Stobutzki and Bellwood, 1997; Faria *et al.*, 2009), we used raceways with rounded, instead of rectangular, bottoms and we doubled the length of the flow straightener to improve the flow dynamics (i.e. create more homogenous flow profiles). We compared the flow velocities between the original and our updated design at three raceway sections (40 mm from the start and the centre and 40 mm before the end; see Fig. 3). In detail, we quantified the modelled flow velocities and turbulence intensity (data not shown) in each raceway at the three sections using 2×2-mm grids. We then compared flow velocity within and between the chamber designs using statistical models (see statistical analyses).

### Experimental testing

#### Animal husbandry

All fish rearing and experimentation was conducted in September 2019 under animal ethics approval from the Animal Ethics Committee at James Cook University (Animal Ethics Permit A2473). Breeding pairs of the cinnamon anemonefish (*A. melanopus*) held in captivity for several years were originally collected from the Great Barrier Reef by professional divers (Cairns Marine, Cairns, Australia). Breeding pairs were maintained outdoors in 80-L tanks at the Marine and Aquaculture Research Facility at James Cook University (Townsville, Australia). Egg clutches that were laid on half a terracotta pot from one of the breeding pairs were transported to the National Sea Simulator (SeaSim) at the Australian Institute of Marine Science two nights before hatch. Here, the pot with the egg clutch was deployed in a rectangular 50-L tank with gentle water flow directed on the clutch (0.3 L min^−1^, 28.5°C, 35 ppt salinity). After seven nights, the larvae hatched (90% hatching success) and the fish were supplied with rotifers (*Brachionus plicatilis*) enriched overnight (Selco© S.parkle, concentrate, Inve Aquaculture Inc., Salt Lake City, USA) at a concentration of 15 rotifers mL^−1^. From 5 days post-hatch (dph), freshly hatched Artemia nauplii (*Artemia salina*) were added to the tanks (2 artemia mL^−1^, Inve Aquaculture Inc., Salt Lake City, USA).

Barramundi (*L. calcarifer*) larvae were supplied by Jarrod Guppy and Adrien Marc (Centre for Sustainable Tropical Fisheries and Aquaculture, James Cook University). After injection with luteinizing hormone-releasing hormone analogue, 12 broodstock barramundi (4 females and 8 males) underwent communal mass spawning as described in Thépot and Jerry (2015). After hatching (14 hpf; average hatch tank conditions: 30°C, 8.1 pH, 30 ppt salinity, >5.0 mg L^−1^ O_2_), larvae were transferred to the SeaSim. Here, the barramundi larvae were maintained in rectangular 50-L flow-through tanks (28.5°C, 35 ppt salinity). Barramundi larvae were reared in ‘green water’ (0.5 × 10e6 cells mL^−1^, Nanno 3600™, Reed Mariculture Inc., CA, USA) until 7 dph and were provided with enriched rotifers(0–16 dph, 15 rotifers mL^−1^) and freshly hatched artemia(2 artemia mL^−1^) from 12 dph onwards. In total, cinnamon anemonefish and barramundi larvae were reared over 3 weeks.

#### Swimming protocols

**Figure 4:**
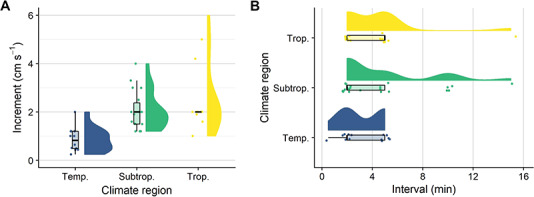
Overview of critical swimming speed (*U*_crit_) protocols used for testing the swimming performance of early life stages of marine fishes. During a *U*_crit_ protocol, the water velocity is continuously increased in pre-defined increments (step heights) and intervals (step lengths) until the tested animal fatigues. (**A**) Increments and (**B**) intervals are separated by the temperate, subtropical and tropical climate regions the tested species inhabit (blue, green and yellow, respectively). Data on *U*_crit_ protocols were collected through a systematic literature search (see Downie *et al.*, 2020 for the search term), and 36 papers were identified (*n* = 56 trials). We excluded one polar species (shorthorn sculpin; 1-cm s^−1^ increment, 2-min interval). Raw data, summary statistics and probability density were visualized using raincloud plots (Allen *et al.*, 2019).

Protocols for measuring critical swimming speeds (*U*_crit_) consist of a step-wise increase in flow velocity until the test subject fatigues. Protocols can vary in the step height (increment; cm s^−1^) or step length (interval; min) and various combinations have been used for measuring *U*_crit_ (see Downie *et al.*, 2020 for a review of *U*_crit_ criteria). Most protocols are adapted to the swimming abilities of the tested species and vary across climate regions (see Fig. 4 for a systematic overview of protocols used for testing *U*_crit_ in early life stages of marine fishes). In the current experiment, we used the newly developed swimming chambers and the automated control settings to investigate how different protocols affect *U*_crit_ estimates for early life stages of two tropical fishes, the coral reef cinnamon anemonefish and the catadromous barramundi. We determined the *U*_crit_ of both species at comparable body lengths (ca. 4–10 mm SL) and swam cinnamon anemonefish at 4, 8, 12 and 16 dph and barramundi at 12, 16 and 20 dph. The protocols differed slightly between the two species, as barramundi larvae are weaker swimmers and could not be exposed to the same increments (i.e. step heights) as used for the anemonefish offspring (B. Illing, pers. obs.). Therefore, the protocols consisted of increments of 1.0, 1.5 and 2.0 cm s^−1^ for cinnamon anemonefish and 0.25, 0.50 and 1.00 cm s^−1^ for barramundi. For both species, we used 2-, 5- and 10-min intervals; an additional 20-min interval was also used for cinnamon anemonefish. These settings allowed us to make comparisons with previously used protocols, for example, those used with cinnamon anemonefish (Fisher *et al.*, 2000; Green and Fisher, 2004). We calculated *U*_crit_ following Brett (1964) as

$$ {U}_{\mathrm{crit}}=U+\Big(t\times{t_{\mathrm{i}}}^{-1}\times{U}_{\mathrm{i}}\Big), $$

where *U* is the penultimate speed a fish was able to maintain, *U*_i_ is the respective velocity increment (0.25, 0.50, 1.00, 1.50 or 2.00 cm s^−1^), *t* is the time spent swimming at the final velocity increment and *t*_i_ is the respective time interval for each velocity increment (2, 5, 10 or 20 min). We considered fish to have reached their *U*_crit_ when they could not maintain position for a full time interval (Fisher *et al.*, 2005). Although several fish are routinely swum at the same time in multi-lane swimming chambers (Faria *et al.*, 2009, 2014), inter-individual differences in performance can lead to situations where some individuals are still swimming and others are already fatigued and forced against the rear grid. In our study, we stopped trials once the last swimming fish became fatigued. For the fish that fatigued before, we calculated the time spent at the grid by subtracting each fish’s time spent swimming from the last swimming fish’s total trial time. This allowed us to assess which of the protocols reduced the time spent at the rear grid after fatigue and aligned best with current animal welfare considerations.

### Statistical analyses

All analyses were performed in R (version 3.5.2) (R Development Core Team, 2018). Data distributions were explored using correlation matrices (package ‘GGally’, v. 1.5.0) (Schloerke, 2020). We used a Bayesian framework to obtain distributions of the modelled flow velocities and to better estimate the variation within and between the two designs. Frequentist analyses were used for particle tracking and experimental validation, and final models were selected using the Akaike information criterion (AIC) for small sample sizes (‘AICc’, package ‘MuMIn’, v. 1.0.0) (Barton, 2009). For the flow modelling, Bayesian generalized linear models were created for both 10 and 40 cm s^−1^ set velocities (package ‘rstanarm’, v.2.19.3) (Goodrich *et al.*, 2020). We used default, weakly informative priors and a Gaussian likelihood, and we ran diagnostics on auto-correlation and convergence with package ‘coda’ (v.0.19-3) (Plummer *et al.*, 2019). Candidate models were compared using expected log predictive density using the ‘loo_compare’ function (package ‘loo’, v.2.3.1) (Vehtari *et al.*, 2020). The two final models (velocity_modeled ~ chamb_design+chamb_section) were executed with three chains and fully converged using a warm-up of 1000 and a total of 10 000 iterations. Prior and posterior distributions were examined with the function ‘pp_check’ (package ‘bayesplot’, v.1.7.1) (Gabry and Mahr, 2020). Using the packages ‘coda’ and ‘emmeans’ (v. 1.4.2) (Lenth, 2020), we then simulated data from the posterior predictive distribution and we obtained estimated marginal means and highest posterior density (HPD) intervals.

For the particle tracking and experimental validation data, (generalized) linear regression models were created. The exception was for the cinnamon anemonefish trials where a generalized additive model best explained the observed variance (package ‘mgcv’, v. 1.8–28) (Wood, 2019). The most parsimonious model based on the AIC was selected. Diagnostic plots helped verify normality and homogeneity of variance of the model residuals (i.e. *Q*–*Q* plots and residual versus fitted plots) (package ‘car’, v. 3.0–3) (Fox and Weisberg, 2019). Post-hoc comparisons and predictions from the (generalized) linear regression models were made using the package ‘emmeans’, whereas pairwise comparisons of the generalized additive model outcome were undertaken with manually created contrast matrices using the ‘glht’ function (package ‘multcomp’, v. 1.4-10) (Hothorn *et al.*, 2020). Data were visualized with packages ‘tidyverse’ (v. 1.3.0.) (Wickham, 2019), ‘cowplot’ (v. 1.0.0) (Wilke, 2019) and ‘magick’ (v. 2.3) (Ooms, 2020).

## Results

### Automated flow control

The algorithm successfully regulated and monitored the flow control (see Supplementary Material S2 for an overview of the programmable logic control and the code). Several warm-up trials were required to predict the correct settings for positioning the two different-sized ball-pen valves that regulated flow velocity. Thereafter, set and measured flow velocities fully aligned (Fig. 2A) and the algorithm created reproducible flow velocities, irrespective of the time intervals used (Fig. 2B).

### Flow dynamics: particle tracking

In the newly designed chambers, uniform and straight flow was observed (see https://doi.org/10.5061/dryad.z34tmpgb7 for all data and exemplary Flowtrace visualizations at 10 and 40 cm s^−1^, respectively). No differences in flow velocity were observed between raceways (linear model, *P* > 0.05, df = 240), and the slope of the linear model explaining the variability in the data best (measured velocity ~ set velocity × raceway) was indistinguishable from the optimal 1:1 line (Fig. 2C). Larger variance was observed at higher velocities (Figs 3 and 2C).

### Flow dynamics: modelling

The modelled flow velocity did not differ between the modified and original swimming chamber design, at neither 10 nor 40 cm s^−1^ (i.e. the 95% HPD intervals intersected with zero). However, the new design was slightly more accurate at the higher velocity (Fig. 3B). The modified design had a median velocity of 40.0 cm s^−1^ (39.3–40.6 cm s^−1^), whereas the original design had a slightly larger median (40.4 cm s^−1^) and range (39.8–41.0 cm s^−1^). No differences in modelled velocity were found across the three tested sections; in other words, in all comparisons, the 95% HPD intersected with zero.

### Experimental testing

**Figure 5:**
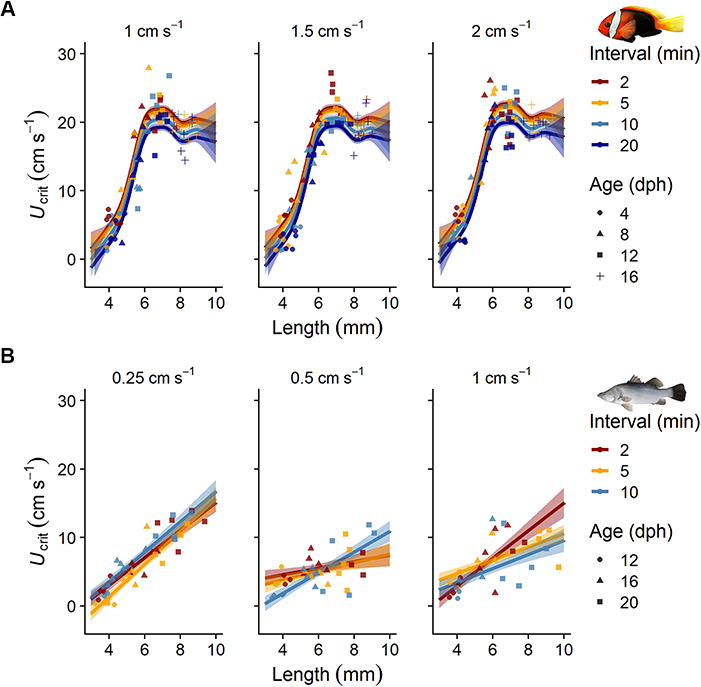
Effect of different increment (step height, sub-panels) and interval (step duration, colour-coded) protocols on critical swimming speed (*U*_crit_) estimates of early life stages of (**A**) cinnamon anemonefish (*Amphiprion melanopus*) and (**B**) barramundi (*Lates calcarifer*). Symbols represent the original *U*_crit_ data (*n* = 5 per increment and interval combination). Symbol shapes indicate the age at which the fishes were tested (in dph). Linear model predictions (mean ± SE) are given for each protocol (see Supplementary Material S3 for the model parameters).

The *U*_crit_ of early life stages of tropical cinnamon anemonefish (16.23 ± 0.39 cm s^−1^, mean ± SE) increased significantly across the first weeks of life (generalized additive model, *P* < 0.001, adjusted R^2^ = 0.86, deviance explained 89.1%, *n* = 236). SL, used as a smooth term in the model, had a significant effect on *U*_crit_ (edf = 6.85, F = 221.8, *P* < 0.001). After metamorphosis and the transition from a pelagic to a benthic lifestyle, *U*_crit_ of cinnamon anemonefish reached a plateau (Fig. 5A). Pairwise comparisons using the *P* value adjustment method between the tested increment protocols were not significant, indicating that increasing the step heights of the *U*_crit_ protocol from 1.0 to 1.5 or 2.0 cm s^−1^ had no effect on the *U*_crit_ of cinnamon anemonefish. However, extending the intervals (i.e. the step length) of the *U*_crit_ protocols by more than 2.5 times significantly decreased *U*_crit_ of cinnamon anemonefish. We found that extending the interval from 2 to 5 min decreased *U*_crit_ on average by (mean ± SE) 0.49 (± 0.39) cm s^−1^ (*P* = 0.69), whereas increasing intervals from 2 to 10 min and 20 min decreased *U*_crit_ significantly by 1.58 (± 0.39) and 2.73 (± 0.39) cm s^−1^, respectively (*P* value adjustment method, *P* < 0.01).

Critical swimming speeds of barramundi offspring were positively correlated with SL, with the best fitting model including interaction terms between SL, increments and intervals (linear model, *P* < 0.001, adjusted R^2^ = 0.62, df = 102). For each millimetre in length, the barramundi increased their *U*_crit_ by 2.00 (± 0.30) cm s^−1^. In contrast to the findings from the anemonefish, longer intervals did not significantly change *U*_crit_ estimates for barramundi. The *U*_crit_ estimates for barramundi that were swum at the medium 0.5-cm s^−1^ increment height (4.93 ± 0.35 cm s^−1^) were lower than in both other treatments (*U*_crit_ of 6.47 ± 0.33 and 6.05 ± 0.37 cm s^−1^ at 0.25 and 1.00 cm s^−1^ increments, respectively) (Fig. 5B). Tukey post-hoc tests identified the difference between the 0.25- and 0.50-cm s^−1^ increments as significant (*P* < 0.01, df = 102) but not the other combinations.

**Figure 6:**
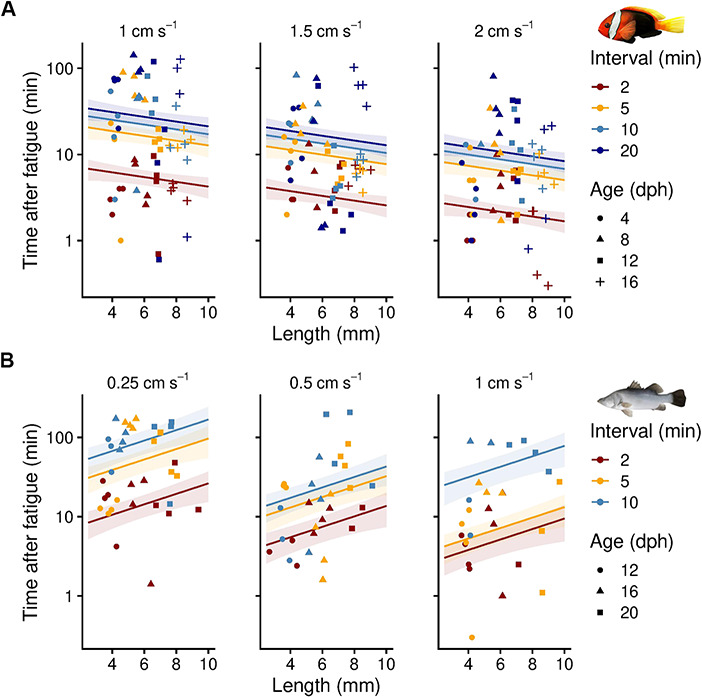
Effect of different increment (step height, sub-panels) and interval (step duration, colour-coded) protocols on the time that fishes, fatigued from a *U*_crit_ trial (Fig. 5), spent resting at the rear grid of the swimming chamber before trials were stopped. Linear model predictions (mean ± SE) are given for each protocol (see Supplementary Material S3 for model parameters) using early life stages of (**A**) cinnamon anemonefish (*Amphiprion melanopus*) and (**B**) barramundi (*Lates calcarifer*). Symbols represent raw data (*n* = 2–4 per increment and interval combination) and shapes indicate the age at which the fishes were tested (in dph). Please note the logarithmic scaling of the y-axes.

The time that fatigued fishes spent at the rear grid before the trials were stopped slightly decreased with size in cinnamon anemonefish (linear model, *P* = 0.21, adjusted R^2^ = 0.30, df = 175); however, this trend was reversed in barramundi, where larger, fatigued individuals spent significantly more time at the rear grid than smaller, fatigued conspecifics (linear model, *P* = 0.02, adjusted R^2^ = 0.41, df = 83) (Fig. 6). Faster trials, i.e. when swimming protocols with higher increments and shorter intervals were used, led to a significantly shorter time that fatigued fish spend at the rear grid. This was observed in both cinnamon anemonefish (Tukey post-hoc tests, *P* < 0.01, df = 175) and barramundi (Tukey post-hoc tests, *P* < 0.01, df = 83) (see Fig. 6 and Supplementary Material S3 for model parameters).

## Discussion

Swimming performance is a critical metric for estimating locomotory capacity in fishes, and an important proxy for estimating the effects of environmental stress. Yet, most swimming chambers require manual control of water flow and water quality parameters. In this study, we improved the accuracy and repeatability of swimming performance measurements by automating multi-lane swimming chambers for small fishes with an open-source computer algorithm. Using CFD software, we found that structural modifications to an original swimming chamber design reduced areas of lower flow but only slightly increased the accuracy of flow velocity at higher speeds. Experimental particle-tracking results in the modified swimming chamber confirmed the modelled uniform water flow. We used the automated chambers to test critical swimming speeds (*U*_crit_) of early life stages of two tropical fishes and found species-specific sensitivity to changes in swimming protocols.

### Swimming chamber design

In swimming chambers, fishes can save substantial amounts of energy by swimming close to wall sections where viscosity restricts the velocity gradient of water (Brett, 1964; van den Thillart *et al.*, 2004; Ellerby and Herskin, 2013). In addition, the size and structure of the chambers can affect flow conditions and a study on small-bodied or juvenile Australian freshwater fish found this to affect *U*_crit_ estimates (Kern *et al.*, 2018). Given the substantial reduction in power requirements for fishes to swim in areas of lower flow velocity, we designed the new multi-lane swimming chamber with convex instead of rectangular raceways. This modification reduced the areas of lower flow velocity while still allowing future recording of distortion-free, high-angle videos of experimental animals. Changing the raceways design was in line with previous findings on the effects of experimental setups on the swimming performance of small fishes. In a study on post-smolt Atlantic salmon (*Salmo salar*), Hvas and Oppedal (2019) found that a rectangular swimming chamber design, and associated irregularities in the flow profile, could have contributed to lower critical swimming speeds (*U*_crit_) of Atlantic salmon when compared with a design with circular cross-sections. A potential caveat of our study is that we could not directly compare *U*_crit_ estimates between the rectangular and rounded raceway designs. However, fishes can actively avoid areas of high flow (e.g. the central cross-section of a raceway) (Tudorache *et al.*, 2013), which supports our decision to remove the bottom corners and generate potentially more conservative *U*_crit_ estimates. Another important modification in our study was the elongation of the flow straightener, a structure consisting of aligned tubes to minimize turbulence and velocity gradients at the front section of the raceways. Most fishes are tested in swimming chambers with rectilinear and uniform flow conditions, as turbulence intensity may affect fish swimming performance and energetics in various ways (Cotel and Webb, 2015; van der Hoop *et al.*, 2018). Optimal flow straightener length to size proportions can be found at 6 or 8 (Bell and Terhune, 1970). As flow profiles in pipes need some time to develop, a certain hydrodynamic entrance length must be considered. When turbulent flow enters pipes, such as individual flow straightener tubes, this entrance effect becomes negligible when the pipe length is roughly 10 times larger than the pipe diameter (Çengel and Cimbala, 2006). In the case of the multi-lane swimming chambers, this critical flow straightener length is reached at 50 mm. Thus, doubling the flow straightener length from 40 to 80 mm helped develop the full, rectilinear flow profile.

### Automated flow control

Automation and control of fish swimming chambers has developed in conjunction with digital advances and available computer technology (Gehrke *et al.*, 1990). Today, control and monitoring of environmental conditions and fish performance in swimming chambers is readily available (e.g. automated measurements in intermittent-flow respirometry). Still, most systems lack a computerized control of water flow and few commercial software exists to automate and control water velocity in swim tunnels (e.g. AutoSwim, Loligo® Systems, Viborg, Denmark). Although our approach requires the use of industrial, micro-PLCs and supervisory control and data acquisition (SCADA) systems, we provide a first open-source algorithm for automating and controlling water velocity in multi-lane swimming chambers. Here, the benefits of the automated swimming chambers are (i) a reduced interference between the operator and the system and potentially less-impacted swimming performance measurements; (ii) more accurate adjustments of flow velocity, i.e. removal of potential delays and imprecise valve adjustments; and (iii) the ability to test more fishes at the same time. We acknowledge the limited availability of commercial SCADA systems and industrial-level micro-PLCs to many researchers; however, some open-source software alternatives and low-cost hardware solutions exist that can make use of the algorithm’s logic (Supplementary Material S2). For example, the OpenPLC Project (https://openplcproject.com) offers an open-source SCADA system, an editor to create ladder logic and function blocks and a software to control several embedded system platforms (e.g. Raspberry Pi, FreeWave and UniPi). Other low-cost microcontroller platforms, for example Arduino models, can be controlled through an independent development environment using desktop computers. In fact, a recent study made use of these latter components to automate a swimming flume for individual zebrafish (Widrick *et al.*, 2018). Regarding the algorithm’s performance, some of the observed variability in measured versus set velocity (Fig. 2B) can be explained by the electrical actuators’ hysteresis (about 4° at any set angle). Using more than two proportional control valves (Fig. 1) could further reduce this variability but would also increase the setup’s technical complexity. The algorithm could further be used in aquaculture engineering to control and automate water flow patterns and exercise fishes to improve growth and fitness. In fact, exercising fishes in aquaculture has been shown to improve growth and optimal rates have been observed at speeds where energetic efficiency is highest (Palstra and Planas, 2011; Palstra *et al.*, 2019). Future work could further help remove observer bias and operator presence by remotely operating the swimming chambers using machine learning and remotely monitoring fish performance, pose and position (Graving *et al.*, 2019).

### Flow dynamics

A combination of CFD with non-intrusive experimental validation (e.g. particle tracking) has been considered valuable for assessing flow dynamics in biophysical research (Fiore *et al.*, 2017; Divi *et al.*, 2018). Here, we used a CFD software and particle tracking of fluorescent, neutrally buoyant microspheres to validate flow velocity and uniformity in the updated design of the multi-lane swimming chamber. The results from the CFD models showed no significant differences in flow velocity or turbulence intensity (data not presented) between the original and updated design with rounded raceways and elongated flow straightener. The small differences in flow velocity across designs might be dispensable when considering the observed inter-individual variability in swimming performance in early life stages of cinnamon anemonefish and barramundi. Although we could not directly compare swimming performance between the two different multi-lane swimming chamber designs, body size and used swimming protocols could have more pronounced effects than minute differences in flow velocity (Fig. 5). Still, removing some areas of lower flow velocity (i.e. the bottom corners of the raceways) helped create more uniform flow patterns. This reduced options for the tested fishes to escape the higher flow velocity in the central cross-sections ensuring they were really swimming against the set velocity. Some non-intrusive experimental techniques estimate flow rate by measuring flow velocities with the Doppler shift induced in reflected sound or light energy by buoyant particles (e.g. acoustic or laser Doppler velocimetry) (Çengel and Cimbala, 2006; Ellerby and Herskin, 2013). Other methods, such as particle image or tracking velocimetry, use laser sheets at cross-sectional areas to track particles. However, most of these techniques are difficult to implement and optimize, which is why open-source algorithms such as Flowtrace, can help visualize flow patterns in a more practicable way (Gilpin *et al.*, 2017). We found Flowtrace to be a cost-effective alternative for experimentally validating flow velocities in swimming chambers. In contrast to other calibration techniques, such as dyes (Kroon and Housefield, 2003), the use of fluorescent microspheres and subsequent Flowtrace visualizations highly improved our qualitative analysis of flow uniformity and velocity. In fact, we were able to confirm the CFD findings on flow velocity and observed higher variability in flow velocity at higher set speeds. The CFD findings on turbulence intensity and the 2D-Flowtrace visualizations showed that turbulence was highest just after the flow straightener and the following few centimetres.

### Swimming protocols

Critical swimming speed (*U*_crit_) is often used to define prolonged swimming capabilities of fishes. Although most fishes do not routinely swim at *U*_crit_, the metric is often used as a proxy for condition and larval dispersal (Faria *et al.*, 2011a; Nanninga and Manica, 2018). Critical swimming speeds increase rapidly with larval development (Moyano *et al.*, 2016; Cominassi *et al.*, 2019). However, the swimming performance of many marine demersal fish levels off after metamorphosis and shifts in habitat, e.g. settlement to rocky reefs or tropical coral reefs (Stobutzki and Bellwood, 1994; Leis, 2010, 2011). This change in performance is likely related to physiological and morphological changes associated with the transition from a pelagic to a benthic lifestyle (Nilsson *et al.*, 2007). In line with findings from other pomacentrids (Stobutzki and Bellwood, 1994), the tested cinnamon anemonefish reduced their swimming performance upon metamorphosis and settlement. In contrast, the estuarine barramundi’s swimming performance increased linearly with larval development. However, catadromous barramundi show a similar decrease in locomotor performance once they metamorphose into benthic juveniles, change habitat and become ambush predators (Edmunds *et al.*, 2010). In our study, all tested barramundi were still in the larval phase, though, as metamorphosis occurs in barramundi at about 11 mm (Barlow *et al.*, 1995).

The *U*_crit_ methodology has been criticized for several reasons, including its sensitivity to the time intervals and velocity increments used (Farlinger and Beamish, 1977; Kolok, 1999; Fisher and Leis, 2010; Downie *et al.*, 2020). Although some fish species and life stages seem to be robust against changes in *U*_crit_ protocols (Fisher and Leis, 2010; Dalziel and Schulte, 2012), other findings suggest sensitivities to changes in the methodology (Kolok, 1999; Downie and Kieffer, 2017). No systematic tests have so far been conducted to investigate the effects of development, body length, time intervals and velocity increments on *U*_crit_ of marine fish larvae. Two previous studies on settlement stages of tropical coral reef fishes investigated the effects of different time intervals. Both studies found no differences in *U*_crit_ when individuals were swum at 2 or 5 min (Fisher *et al.*, 2005) and at 2 or 15 min (Hogan *et al.*, 2007) time intervals. Similarly, we found no differences in *U*_crit_ of barramundi offspring that were swum at different time intervals (2, 5, and 10 min). In contrast, *U*_crit_ estimates for the cinnamon anemonefish were significantly affected when time intervals were extended by more than 2.5 times (i.e. from 2 to 10 or 20 min). Swimming speeds and velocity increments are often expressed in relative terms (body lengths per second, BL s^−1^), and a commonly used velocity increment is 3 BL s^−1^ (Bellwood and Fisher, 2001; Fisher, 2005; Fisher *et al.*, 2005). In line with this, we chose similar absolute velocity increments for cinnamon anemonefish (1.0, 1.5 and 2.0 cm s^−1^; circa 4–10 mm SL; min. and max. range 1–5 BL s^−1^), but we had to use lower velocity increments for barramundi offspring, as they exhibited reduced swimming capabilities (0.25, 0.5 and 1.0 cm s^−1^; circa 4–10 mm SL; min. and max. range: 0.25–1 BL s^−1^). In contrast to the above-mentioned studies on the swimming performance of settlement stages of tropical coral reef fishes, our findings suggest species-specific sensitivities of early life stages of tropical fishes to different *U*_crit_ protocols.

Differences in performance across species and life stages should also be considered from an animal welfare perspective when using multi-lane swimming chambers. When swimming several, small fish in multi-lane chambers simultaneously, the time that fatigued fish spend at the rear grid before trials can be ceased should be minimized. Based on our chosen protocols and life stages, we recommend swimming barramundi and cinnamon anemonefish in multi-lane chambers using 5-min intervals and 0.5 and 2.0 cm s^−1^ increments, respectively. Technical modifications, such as openings in the lid on top of each lane, could further assist with quickly removing fatigued fish; however, manipulating the multi-lane swimming chambers while some individuals are still swimming could affect ongoing swimming performance measurements. An alternative is to use single lanes only (Silva *et al.*, 2015) but multi-factorial experiments often require large sample sizes and thus high throughput and simultaneous measurements of several fish (Silva *et al.*, 2016). For other species, we recommend quantifying effects of different velocity increments and time intervals in pre-trials before experimentation to assist comparative studies in accounting for potential biases in methodology. This may be particularly important if the swimming performance data are subsequently used for parameterizing individual-based models that help predict growth and dispersal dynamics of early life stages of fishes.

### Relevance of larval fish swimming performance for conservation

From a conservation perspective, connectivity estimates of fish populations can contribute to improve management efforts, e.g. through assessing marine reserve performance (Harrison *et al.*, 2020). Information on how environmental parameters affect larval fish development and performance are thus important for parameterizing predictive models assessing the growth and dispersal of larvae. Laboratory experiments can assist with creating this information and can simulate current and predicted environmental conditions. With regard to temperature, the development of marine larval stages quickens and dispersal is generally considered to be reduced under warmer conditions in tropical species (Álvarez-Noriega *et al.*, 2020). Indeed, cinnamon anemonefish larvae have been found to have faster development and growth at elevated temperatures, which also increased their critical swimming speeds (Green and Fisher, 2004). To the best of our knowledge, no information exists on how temperature affects swimming in larval barramundi; however, larval barramundi develop faster at elevated temperatures and exhibit higher critical swimming speeds as juveniles (Carey and Franklin, 2009). Integrating this physiological information and the inter-individual variability to individual-based growth and dispersal models will support the robustness of predictions. Currently, the pelagic larval duration is often used to assess the effects of global warming on the individual dispersal capabilities of larval fishes (Kendall *et al.*, 2016). Integrating more accurate findings of larval fish swimming performance under conditions predicted for mid- and end-of-century conditions will strengthen these model predictions and support conservation efforts, such as assessing the replenishment of fish populations and designing future marine protected areas (Álvarez-Romero *et al.*, 2018).

##  

In this study, we updated and automated multi-lane swimming chambers for small fishes. We developed an open-source algorithm to control flow velocity and thereby reduce operator interference and flow inaccuracies that are typically associated with manual control. We compared the swimming chambers’ flow dynamics across designs using CFD software and verified flow homogeneity with experimental particle tracking in the updated chambers. In trials with offspring from two tropical fish species, we tested the automated setups and found that longer critical swimming speed (*U*_crit_) protocol intervals significantly affected *U*_crit_ estimates for cinnamon anemonefish but not for larval barramundi. Integrating the automated swimming test protocols with a computerized control of water parameters (e.g. combined temperature and *p*CO_2_) will facilitate more accurate and repeatable experimentation under changed environmental conditions. Thus, the updated setup enables researchers to collect further information on how individual and co-occurring environmental stressors affect the swimming performance of fishes. This will help parameterize model predictions more accurately and assist in better assessing the effects of combined anthropogenic stressors on fish population dynamics (e.g. population connectivity).

## Supplementary Material

Supp_material_S1_coaa131

Supp_material_S2_coaa131

Supp_material_S3_coaa131
