# Supplementary material for: Automated flow control of a multi-lane swimming chamber for small fishes indicates species-specific sensitivity to experimental protocols
Source: Conserv Physiol. 2021 Jan 7;9(1):coaa131. doi: 10.1093/conphys/coaa131 (PMC7905161; doi:10.1093/conphys/coaa131)
Supplement: Supp_material_S1_coaa131 [file supp_material_s1_coaa131.pdf]

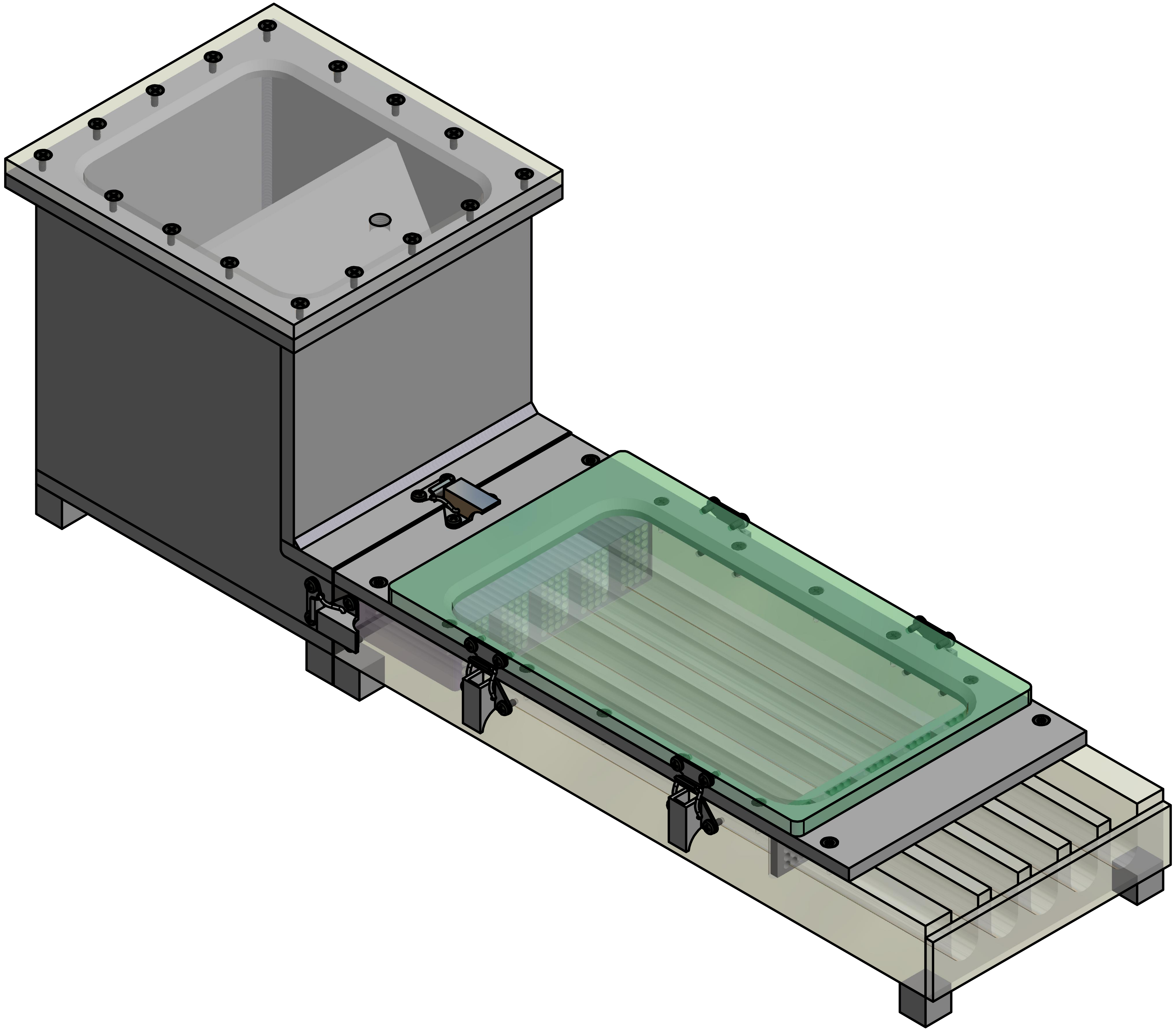

Scale: .4	Designed by njeeves	Checked by	©2016 Australian Institute of Marine Science	Date: 14/08/2020
Material:	Australian Government / Australian Institute of Marine Science		Laval Laminar flow complete.iam	Sheet A3 1 of 6

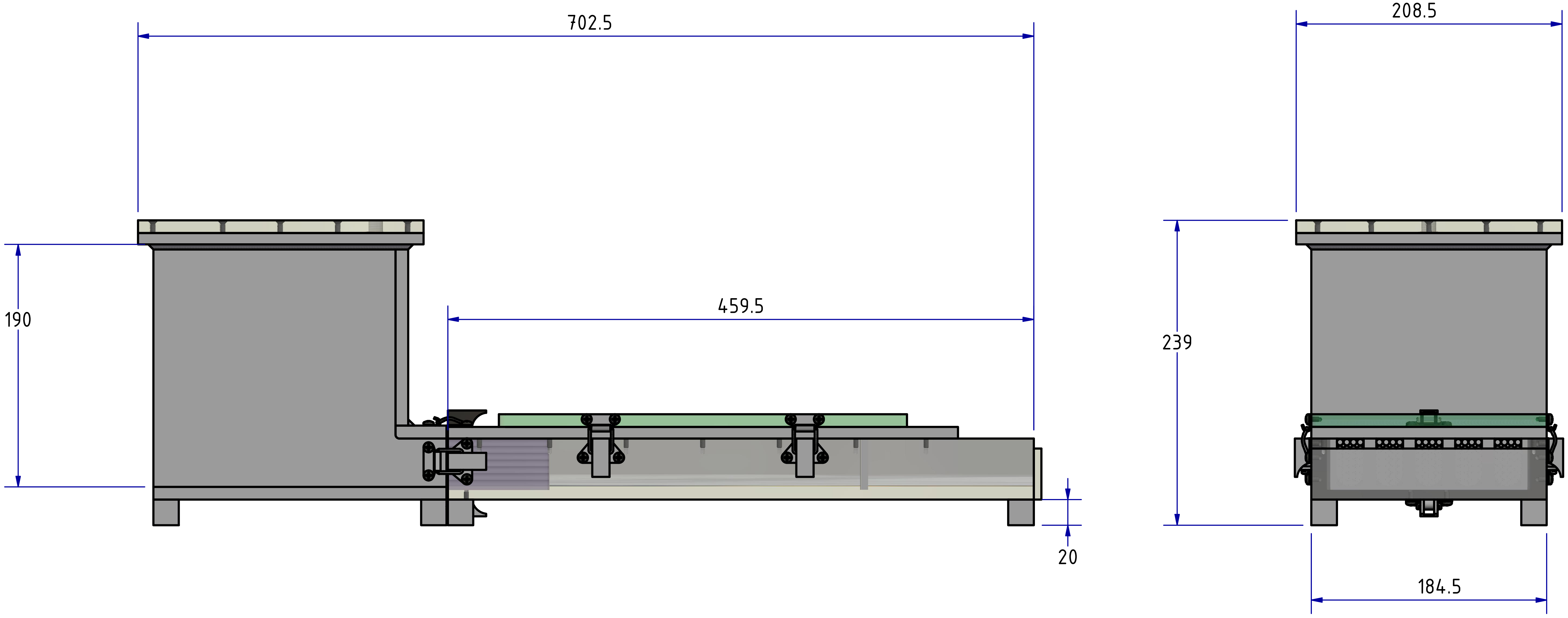

Scale: .3	Designed by njeeves	Checked by	©2016 Australian Institute of Marine Science	Date: 14/08/2020
Material:	Australian Government / Australian Institute of Marine Science		Laval Laminar flow complete.iam	Sheet A3 2 of 6

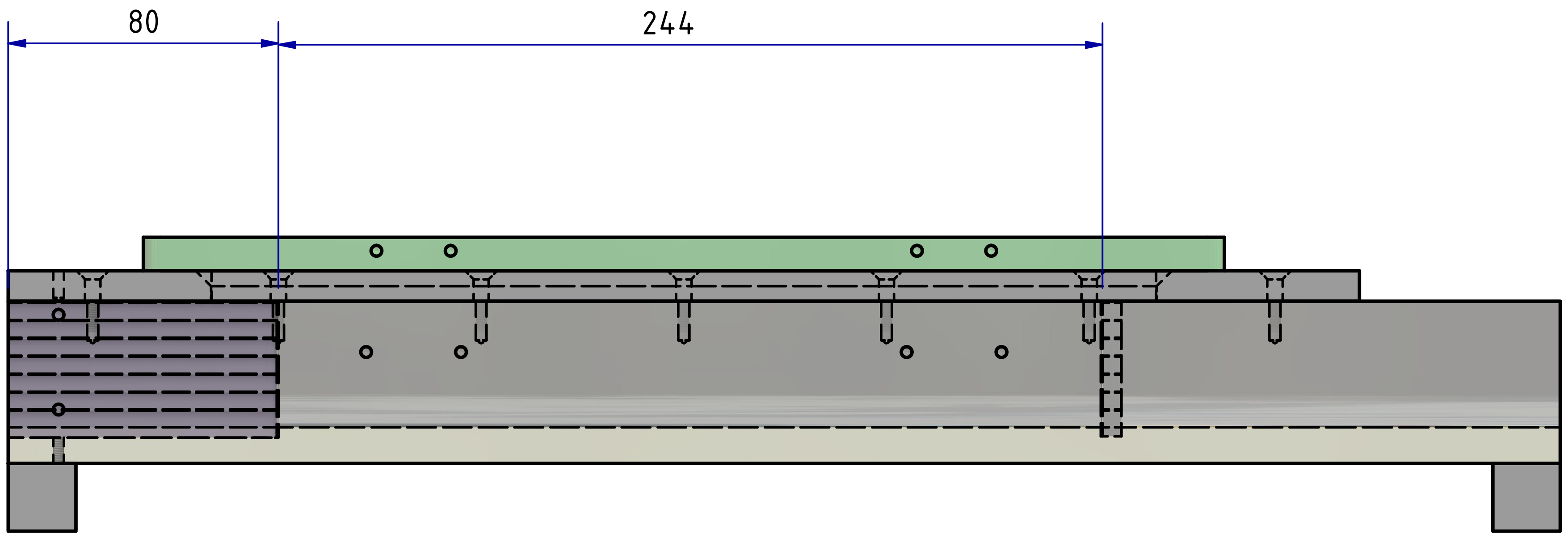

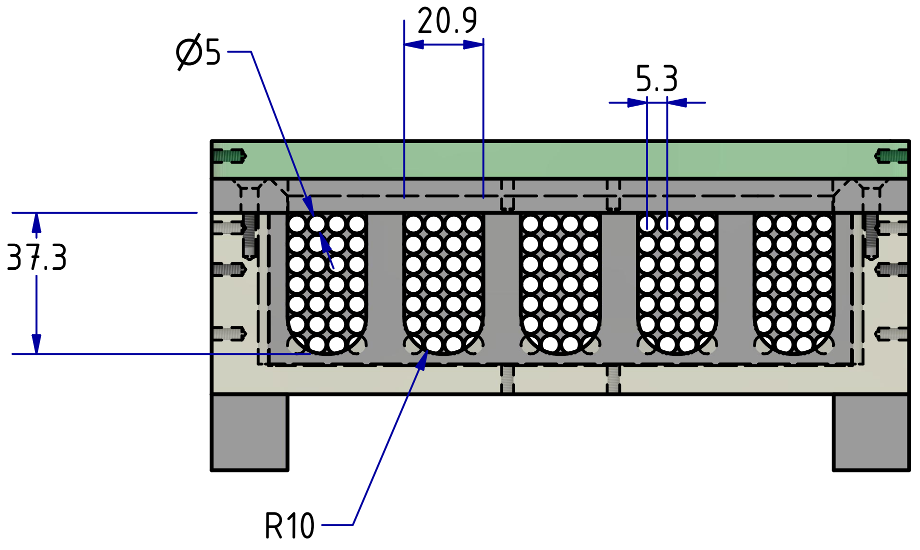

PARTS LIST			
ITEM	QTY	PART NUMBER	DESCRIPTION
1	1	Laminar flow linear block_Shrinkwrap_1	
2	1	Channel Base	
3	1	retaining screen	
4	4	Foot	
5	1	Channel top plate	
6	1	Channel lid	

Scale: .5	Designed by njeeves	Checked by	©2016 Australian Institute of Marine Science	Date: 14/08/2020
Material:			Channel Complete.iam	Sheet A3 3 of 6

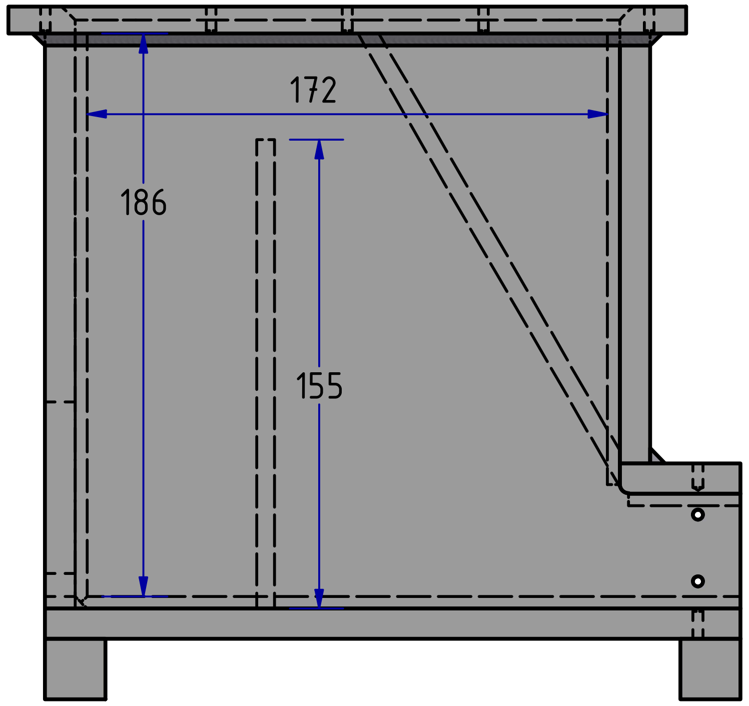

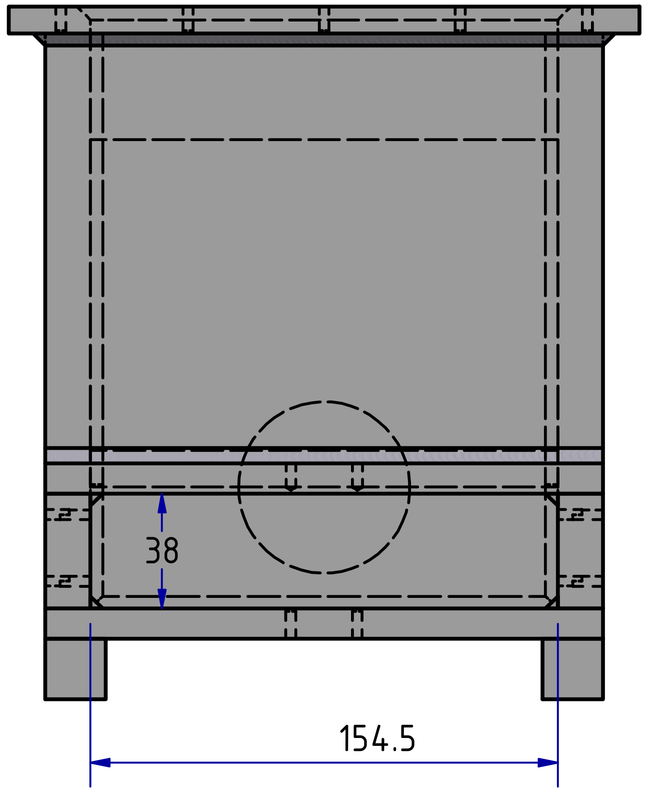

Scale: .4	Designed by njeeves	Checked by	©2016 Australian Institute of Marine Science	Date: 14/08/2020
Material:			Tank welded.iam	Sheet A3 4 of 6

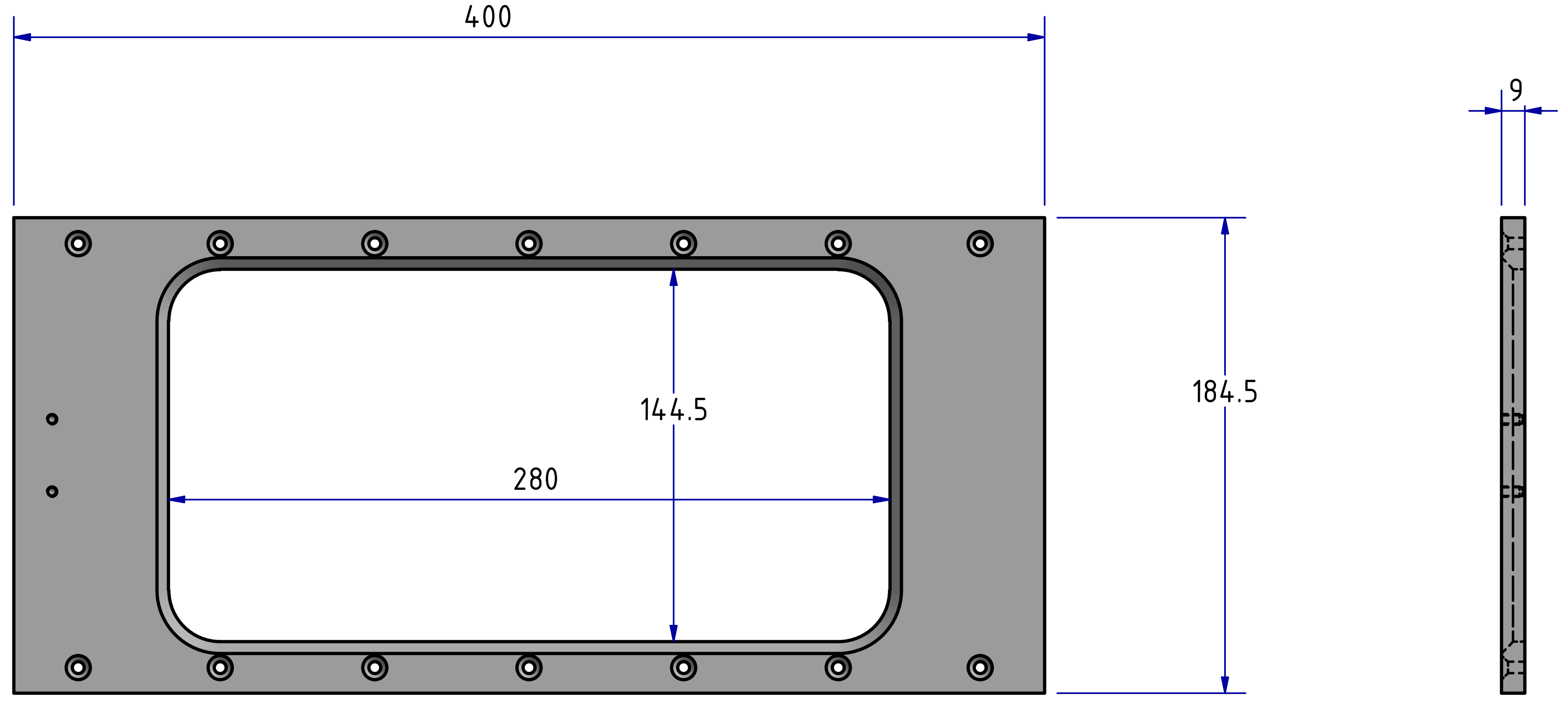

Scale: .4	Designed by njeeves	Checked by	©2016 Australian Institute of Marine Science	Date: 14/08/2020
Material:	Australian Government / Australian Institute of Marine Science		Channel top plate.ipt	Sheet A3 5 of 6

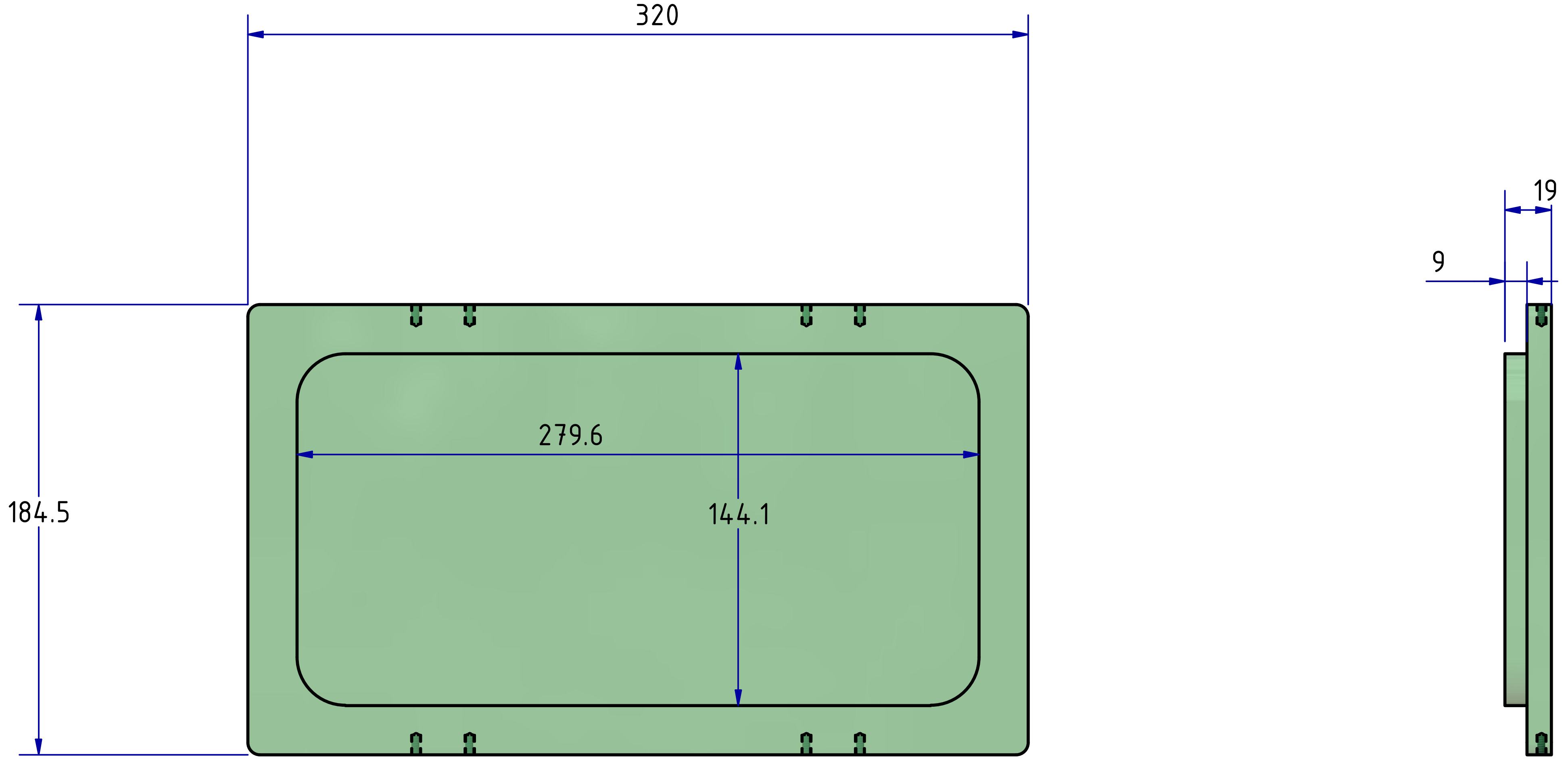

Scale: .4	Designed by njeeves	Checked by	©2016 Australian Institute of Marine Science	Date: 14/08/2020
Material:	Australian Government / Australian Institute of Marine Science		Channel lid.ipt	Sheet A3 6 of 6
