# Supplementary material for: Automated flow control of a multi-lane swimming chamber for small fishes indicates species-specific sensitivity to experimental protocols
Source: Conserv Physiol. 2021 Jan 7;9(1):coaa131. doi: 10.1093/conphys/coaa131 (PMC7905161; doi:10.1093/conphys/coaa131)
Supplement: Supp_material_S2_coaa131 [file supp_material_s2_coaa131.docx]

Supplementary material S2

Illing et al. 2020, Conservation Physiology

Table of Contents

[Overview PLC Logic 1](#_Toc50121710)

[Function blocks 2](#_Toc50121711)

[RampCtl 2](#_Toc50121712)

[RampMPC 12](#_Toc50121713)

# Overview PLC Logic

This document describes how to automate the flow control of swimming chambers using the software [SIMANTIC WinCC V15.1](https://support.industry.siemens.com/cs/document/109761203/delivery-release-wincc-v15-1?dti=0&lc=en-NG). Please note, that essential function blocks need to be added to automate the flow control (e.g., system-specific clocks, scaling of the input and output).


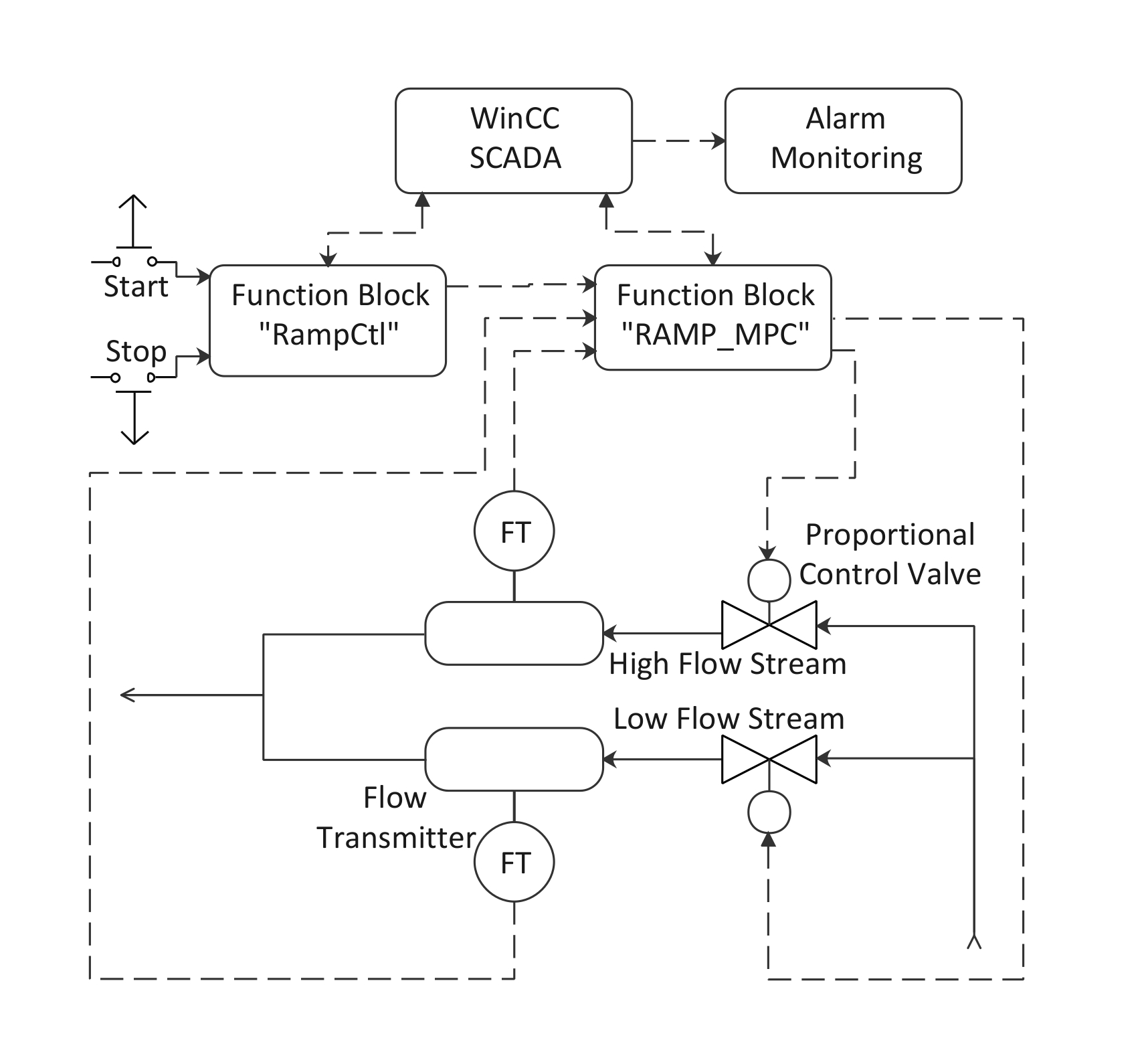


Fig. S2 Overview of the Programmable Logic Control (PLC) for automating flow control in multi-lane swimming chambers.

# Function blocks

The following function blocks are required for automating the flow control.

## RampCtl

In this function block, the following tasks are covered:

1. Gathers the Stage information the user enters into SCADA via the touch screen clients
2. Waits for the Stop/Start signal
3. Depending on how many stages are requested, calculates each stage Start & Finish times
4. Provides feedback to SCADA
5. Provides a Flow Rate Set Value and Start/Stop commands to “RAMP_MPC”

// ***** Function Block - "RampCtl" ***** //

// ** Ramp Stage Control - Flow ** //
(*
Ramp Mater Sequence - "#RM.Seq"
 0: System Idle
 1: Stage 1
 2: Stage 2
 3: Stage 3
 4: Stage 4
 5: Stage 5


*)

// Physical Start Button Pushed - Write Start Command
IF #StartPB THEN
 #RM.Start := TRUE;
END_IF;

// Physical Stop Button Pushed - Write Stop Command
IF #StopPB THEN
 #RM.Stop := TRUE;
END_IF;

// Copy Ramp Master Stages Data
#Stages := #RM."#Stages";

// Stage 0 - System Idle
IF #Stages = 0 THEN
 RETURN;
END_IF;

// Calculate Total Time Hr/Min/Sec Data - Based on number of Stages
IF #Stages > 0 THEN
 #RM.TotalTime.HOUR := #Rmp1.Duration.HOUR;
 #RM.TotalTime.MIN := #Rmp1.Duration.MIN;
 #RM.TotalTime.SEC := #Rmp1.Duration.SEC;
END_IF;
IF #Stages > 1 THEN
 #RM.TotalTime.HOUR := #RM.TotalTime.HOUR+#Rmp2.Duration.HOUR;
 #RM.TotalTime.MIN := #RM.TotalTime.MIN +#Rmp2.Duration.MIN;
 #RM.TotalTime.SEC := #RM.TotalTime.SEC +#Rmp2.Duration.SEC;
END_IF;
IF #Stages > 2 THEN
 #RM.TotalTime.HOUR := #RM.TotalTime.HOUR + #Rmp3.Duration.HOUR;
 #RM.TotalTime.MIN := #RM.TotalTime.MIN + #Rmp3.Duration.MIN;
 #RM.TotalTime.SEC := #RM.TotalTime.SEC + #Rmp3.Duration.SEC;
END_IF;
IF #Stages > 3 THEN
 #RM.TotalTime.HOUR := #RM.TotalTime.HOUR + #Rmp4.Duration.HOUR;
 #RM.TotalTime.MIN := #RM.TotalTime.MIN + #Rmp4.Duration.MIN;
 #RM.TotalTime.SEC := #RM.TotalTime.SEC + #Rmp4.Duration.SEC;
END_IF;
IF #Stages > 4 THEN
 #RM.TotalTime.HOUR := #RM.TotalTime.HOUR + #Rmp5.Duration.HOUR;
 #RM.TotalTime.MIN := #RM.TotalTime.MIN + #Rmp5.Duration.MIN;
 #RM.TotalTime.SEC := #RM.TotalTime.SEC + #Rmp5.Duration.SEC;
END_IF;

// Convert DTL Data to Hr/Min/Sec
#x_dtl:="Add_DTL_TO_SDTL"(IN_SDTL:=#RM.TotalTime, IN_DTL:=#NULL_dtl);
#RM.TotalTime.HOUR :=#x_dtl.HOUR ;
#RM.TotalTime.MIN := #x_dtl.MINUTE ;
#RM.TotalTime.SEC := #x_dtl.SECOND ;

// Ramp Master - Run - Determine Program Stage Progress
IF #RM.FbkRun THEN

 #StagesR:= INT_TO_REAL(#Stages) * 100.0;

 IF #RM.Seq = 1 THEN
 #progress := INT_TO_REAL(#Rmp1.Progress);
 END_IF;
 IF #RM.Seq = 2 THEN
 #progress := INT_TO_REAL(#Rmp1.Progress)+INT_TO_REAL(#Rmp2.Progress);
 END_IF;
 IF #RM.Seq = 3 THEN
 #progress := INT_TO_REAL(#Rmp1.Progress) + INT_TO_REAL(#Rmp2.Progress) + INT_TO_REAL(#Rmp3.Progress);
 END_IF;
 IF #RM.Seq = 4 THEN
 #progress := INT_TO_REAL(#Rmp1.Progress) + INT_TO_REAL(#Rmp2.Progress) + INT_TO_REAL(#Rmp3.Progress) + INT_TO_REAL(#Rmp4.Progress);
 END_IF;
 IF #RM.Seq = 5 THEN
 #progress := INT_TO_REAL(#Rmp1.Progress) + INT_TO_REAL(#Rmp2.Progress) + INT_TO_REAL(#Rmp3.Progress) + INT_TO_REAL(#Rmp4.Progress)+ INT_TO_REAL(#Rmp5.Progress);
 END_IF;
 #progress := (#progress / #StagesR) * 100;
 #RM.Progress := REAL_TO_INT(#progress);
 #FbkRun := true;
ELSE
 #RM.Progress := 0;
 #Rmp1.Progress := 0;
 #Rmp2.Progress := 0;
 #Rmp3.Progress := 0;
 #Rmp4.Progress := 0;
 #Rmp5.Progress := 0;

END_IF;

// Sequence 0 - Idle
IF #RM.Seq = 0 THEN
 #RM.FbkRun := false;
 #FbkRun := FALSE;
END_IF;

// Start Command - Calculate Finish Time
IF #RM.Start THEN
 #RM.FbkRun := TRUE;
 #Data.StartTime[1] := "General".CPUDTL;
 #Data.FinishTime[1] := "Add_DTL_TO_SDTL"(IN_DTL := #Data.StartTime[1], IN_SDTL := #Rmp1.Duration);
 #RM.Start := FALSE;
 #RM.Seq := 1;
 #RM.Progress := 0;
 #Rmp1.Progress := 0;
 #Rmp2.Progress := 0;
 #Rmp3.Progress := 0;
 #Rmp4.Progress := 0;
 #Rmp5.Progress := 0;
END_IF;

// Stop Command - Reset Sequence
IF #RM.Stop THEN
 #RM.FbkRun := false;
 #RM.Stop := FALSE;
 #RM.Seq := 0;
 #RM.CurrentStage := #RM.Seq;
END_IF;

// Not Running - Exit Program
IF #RM.FbkRun <> TRUE THEN
 RETURN;
END_IF;

// Record Current Stage
#RM.CurrentStage := #RM.Seq;

// Calculate Day
IF #RM.Seq = 0 THEN
 #day := FLOOR((DINT_TO_REAL("RAMP_CALCS".DUR1) / 86400));
END_IF;


// Sequence >= 10
IF #RM.Seq >= 10 THEN

 #Rmp1.Follow := TRUE;

 // Calculate Elapsed Time
 #elapsed := T_DIFF(IN1 := "General".CPUDTL, IN2 := #Data.StartTime[1]);

 // Convert Time to Double Interager - Time Basic
 #dint_elapsed := TIME_TO_DINT(#elapsed);
 #RM.Elapsed := "TimeINT_TO_SDTL"(#dint_elapsed);
 #Rmp1.Elapsed := "TimeINT_TO_SDTL"(#dint_elapsed);

 // Set Flow
 #Rmp1.FlowSV := #Rmp1.ValStart+(#Rmp1.ValFinish*(#RM.Seq-10));
 #RM.FlowSV := #Rmp1.FlowSV;

 // Calculate Time Remaining
 #TimePV := T_DIFF(IN1 := #Data.FinishTime[1], IN2 := "General".CPUDTL);
 #TimePV_I := TIME_TO_DINT(#TimePV);

 // Valves at max - Reset Sequence
 IF #RM.VLgeSV >= 99 AND #RM.VSmlSV >= 99 THEN
 #RM.FbkRun := FALSE;
 #RM.Stop := FALSE;
 #RM.Seq := 0;
 #Rmp1.Follow := FALSE;
 #Data.FinishTime[1] := "Add_DTL_TO_SDTL"(IN_DTL := #Data.FinishTime[1], IN_SDTL := #Rmp1.Duration);
 END_IF;

 // Sequence Time Finished - Increment Sequence
 IF #TimePV_I <= 0 THEN
 #RM.FlowSV := #Rmp1.FlowSV ;
 #RM.Seq:=#RM.Seq+1 ;
 #Data.FinishTime[1] := "Add_DTL_TO_SDTL"(IN_DTL := #Data.FinishTime[1], IN_SDTL := #Rmp1.Duration);
 END_IF;

 // Calculate Time Data - Reamining Time Fraction
 #TempDint := TIME_TO_DINT(T_DIFF(IN1 := #Data.FinishTime[1], IN2 := "General".CPUDTL));
 #TempDuration := TIME_TO_DINT(T_DIFF(IN1 := #Data.FinishTime[1], IN2 := #Data.StartTime[1]));
 #TempDint := REAL_TO_INT((DINT_TO_REAL(#TempDint) / DINT_TO_REAL(#TempDuration)) * 100.0);

 // Progress based on Remaining Time Fraction
 IF #TempDint > 0 THEN
 #Rmp1.Progress := 100 - DINT_TO_INT(#TempDint);
 END_IF;

END_IF;


// Sequence 1
IF #RM.Seq = 1 THEN

 #Rmp1.Follow := TRUE;

 IF #Rmp1.ValFinish > 0 THEN
 #RM.Seq := 10;
 RETURN;
 END_IF;

 // Calculate Elapsed Time
 #elapsed := T_DIFF(IN1 := "General".CPUDTL, IN2 := #Data.StartTime[1]);

 // Convert Time to Double Interager - Time Basic
 #Data.FinishTime[1] := "Add_DTL_TO_SDTL"(IN_DTL := #Data.StartTime[1], IN_SDTL := #Rmp1.Duration);
 #dint_elapsed := TIME_TO_DINT(#elapsed);
 #RM.Elapsed := "TimeINT_TO_SDTL"(#dint_elapsed);
 #Rmp1.Elapsed := "TimeINT_TO_SDTL"(#dint_elapsed);

 // Set Flow SV
 #RM.FlowSV := #Rmp1.ValStart;
 #Rmp1.FlowSV := #Rmp1.ValStart;

 // Calculate Time Remaining
 #TimePV := T_DIFF(IN1 :=#Data.FinishTime[1], IN2 := "General".CPUDTL);
 #TimePV_I := TIME_TO_DINT(#TimePV);

 // Sequence Time Finished - Increment Sequence
 IF #TimePV_I <= 0 THEN
 #RM.FlowSV := #Rmp1.ValFinish;
 #Rmp1.FlowSV := #Rmp1.ValFinish;
 #RM.Seq := 0;
 #RM.FlowSV := #Rmp1.ValFinish;
 #Rmp1.FlowSV := #Rmp1.ValFinish;
 #RM.CurrentStage := #RM.Seq;
 IF #RM.CurrentStage <> #RM."#Stages" THEN
 #RM.Seq := 2;
 #Rmp1.Follow := FALSE;
 #Data.StartTime[2] := "General".CPUDTL;
 ELSE
 #RM.Seq := 0;
 END_IF;
 END_IF;

 // Calculate Time Data - Reamining Time Fraction
 #TempDint := TIME_TO_DINT(T_DIFF(IN1 := #Data.FinishTime[1], IN2 := "General".CPUDTL));
 #TempDuration := TIME_TO_DINT(T_DIFF(IN1 := #Data.FinishTime[1], IN2 := #Data.StartTime[1]));
 #TempDint := REAL_TO_INT((DINT_TO_REAL(#TempDint) / DINT_TO_REAL(#TempDuration)) * 100.0);

 // Progress based on Remaining Time Fraction
 IF #TempDint > 0 THEN
 #Rmp1.Progress := 100-DINT_TO_INT(#TempDint);
 END_IF;

END_IF;


IF #RM.Seq = 2 THEN

 #Rmp2.Follow := TRUE;

 // Calculate Elapsed Time
 #elapsed := T_DIFF(IN1 := "General".CPUDTL, IN2 := #Data.StartTime[1]);
 #Data.FinishTime[2] := "Add_DTL_TO_SDTL"(IN_DTL := #Data.StartTime[2], IN_SDTL := #Rmp2.Duration);
 #dint_elapsed := TIME_TO_DINT(#elapsed);
 #RM.Elapsed := "TimeINT_TO_SDTL"(#dint_elapsed);


 // Convert Time to Double Interager - Time Basic
 #elapsed := T_DIFF(IN1 := "General".CPUDTL, IN2 := #Data.StartTime[2]);
 #dint_elapsed := TIME_TO_DINT(#elapsed);
 #Rmp2.Elapsed := "TimeINT_TO_SDTL"(#dint_elapsed);
 #Rmp2.Elapsed := "TimeINT_TO_SDTL"(#dint_elapsed);

 // Set Flow SV
 #RM.FlowSV := #Rmp2.ValStart;
 #Rmp2.FlowSV := #Rmp2.ValStart;

 // Calculate Time Remaining
 #TimePV := T_DIFF(IN1 := #Data.FinishTime[2], IN2 := "General".CPUDTL);
 #TimePV_I := TIME_TO_DINT(#TimePV);

 // Sequence Time Finished - Increment Sequence
 IF #TimePV_I <= 0 THEN
 #RM.FlowSV := #Rmp2.ValFinish;
 #Rmp2.FlowSV := #Rmp2.ValFinish;
 #RM.Seq := 0;
 #RM.FlowSV := #Rmp2.ValFinish;
 #Rmp2.FlowSV := #Rmp2.ValFinish;
 #RM.CurrentStage := #RM.Seq;
 IF #RM.CurrentStage <> #RM."#Stages" THEN
 #RM.Seq := 3;
 #Rmp2.Follow := FALSE;
 #Data.StartTime[3] := "General".CPUDTL;
 ELSE
 #RM.Seq := 0;
 END_IF;
 END_IF;

 // Calculate Time Data - Reamining Time Fraction
 #TempDint := TIME_TO_DINT(T_DIFF(IN1 := #Data.FinishTime[2], IN2 := "General".CPUDTL));
 #TempDuration := TIME_TO_DINT(T_DIFF(IN1 := #Data.FinishTime[2], IN2 := #Data.StartTime[2]));
 #TempDint := REAL_TO_INT((DINT_TO_REAL(#TempDint) / DINT_TO_REAL(#TempDuration)) * 100.0);

 // Progress based on Remaining Time Fraction
 IF #TempDint > 0 THEN
 #Rmp2.Progress := 100 - DINT_TO_INT(#TempDint);
 END_IF;

END_IF;


IF #RM.Seq =3 THEN

 #Rmp3.Follow := TRUE;

 // Calculate Elapsed Time
 #elapsed := T_DIFF(IN1 := "General".CPUDTL, IN2 := #Data.StartTime[1]);
 #Data.FinishTime[3] := "Add_DTL_TO_SDTL"(IN_DTL := #Data.StartTime[3], IN_SDTL := #Rmp3.Duration);
 #dint_elapsed := TIME_TO_DINT(#elapsed);
 #RM.Elapsed := "TimeINT_TO_SDTL"(#dint_elapsed);

 // Convert Time to Double Interager - Time Basic
 #elapsed := T_DIFF(IN1 := "General".CPUDTL, IN2 := #Data.StartTime[3]); #dint_elapsed := TIME_TO_DINT(#elapsed);
 #Rmp3.Elapsed := "TimeINT_TO_SDTL"(#dint_elapsed);

 // Set Flow SV
 #RM.FlowSV := #Rmp3.ValStart;
 #Rmp3.FlowSV := #Rmp3.ValStart;

 // Calculate Time Remaining
 #TimePV := T_DIFF(IN1 := #Data.FinishTime[3], IN2 := "General".CPUDTL);
 #TimePV_I := TIME_TO_DINT(#TimePV);

 // Sequence Time Finished - Increment Sequence
 IF #TimePV_I <= 0 THEN
 #RM.FlowSV := #Rmp3.ValFinish;
 #Rmp3.FlowSV := #Rmp3.ValFinish;
 #RM.Seq := 0;
 #RM.FlowSV := #Rmp3.ValFinish;
 #Rmp3.FlowSV := #Rmp3.ValFinish;
 #RM.CurrentStage := #RM.Seq;
 IF #RM.CurrentStage <> #RM."#Stages" THEN
 #RM.Seq := 4;
 #Rmp3.Follow := FALSE;
 #Data.StartTime[4] := "General".CPUDTL;
 ELSE
 #RM.Seq := 0;
 END_IF;
 END_IF;

 // Calculate Time Data - Reamining Time Fraction
 #TempDint := TIME_TO_DINT(T_DIFF(IN1 := #Data.FinishTime[3], IN2 := "General".CPUDTL));
 #TempDuration := TIME_TO_DINT(T_DIFF(IN1 := #Data.FinishTime[3], IN2 := #Data.StartTime[3]));
 #TempDint := REAL_TO_INT((DINT_TO_REAL(#TempDint) / DINT_TO_REAL(#TempDuration)) * 100.0);

 // Progress based on Remaining Time Fraction
 IF #TempDint > 0 THEN
 #Rmp3.Progress := 100 - DINT_TO_INT(#TempDint);
 END_IF;
END_IF;

// Sequence 4
IF #RM.Seq = 4 THEN

 #Rmp4.Follow := TRUE;

 // Calculate Elapsed Time
 #elapsed := T_DIFF(IN1 := "General".CPUDTL, IN2 := #Data.StartTime[1]);
 #Data.FinishTime[4] := "Add_DTL_TO_SDTL"(IN_DTL := #Data.StartTime[4], IN_SDTL := #Rmp4.Duration);
 #dint_elapsed := TIME_TO_DINT(#elapsed);
 #RM.Elapsed := "TimeINT_TO_SDTL"(#dint_elapsed);

 // Convert Time to Double Interager - Time Basic
 #elapsed := T_DIFF(IN1 := "General".CPUDTL, IN2 := #Data.StartTime[4]);
 #dint_elapsed := TIME_TO_DINT(#elapsed);
 #Rmp4.Elapsed := "TimeINT_TO_SDTL"(#dint_elapsed);

 // Set Flow SV
 #RM.FlowSV := #Rmp4.ValStart;
 #Rmp4.FlowSV := #Rmp4.ValStart;

 // Calculate Time Remaining
 #TimePV := T_DIFF(IN1 := #Data.FinishTime[4], IN2 := "General".CPUDTL);
 #TimePV_I := TIME_TO_DINT(#TimePV);

 // Sequence Time Finished - Increment Sequence
 IF #TimePV_I <= 0 THEN
 #RM.FlowSV := #Rmp4.ValFinish;
 #Rmp4.FlowSV := #Rmp4.ValFinish;
 #RM.Seq := 0;
 #RM.FlowSV := #Rmp4.ValFinish;
 #Rmp4.FlowSV := #Rmp4.ValFinish;
 #RM.CurrentStage := #RM.Seq;
 IF #RM.CurrentStage <> #RM."#Stages" THEN
 #RM.Seq := 5;
 #Rmp4.Follow := FALSE;
 #Data.StartTime[5] := "General".CPUDTL;
 ELSE
 #RM.Seq := 0;
 END_IF;
 END_IF;

 // Calculate Time Data - Reamining Time Fraction
 #TempDint := TIME_TO_DINT(T_DIFF(IN1 := #Data.FinishTime[4], IN2 := "General".CPUDTL));
 #TempDuration := TIME_TO_DINT(T_DIFF(IN1 := #Data.FinishTime[4], IN2 := #Data.StartTime[4]));
 #TempDint := REAL_TO_INT((DINT_TO_REAL(#TempDint) / DINT_TO_REAL(#TempDuration)) * 100.0);

 // Progress based on Remaining Time Fraction
 IF #TempDint > 0 THEN
 #Rmp4.Progress := 100 - DINT_TO_INT(#TempDint);
 END_IF;

END_IF;

// Sequence 5
IF #RM.Seq = 5 THEN

 #Rmp5.Follow := TRUE;

 // Calculate Elapsed Time
 #elapsed := T_DIFF(IN1 := "General".CPUDTL, IN2 := #Data.StartTime[1]);
 #Data.FinishTime[5] := "Add_DTL_TO_SDTL"(IN_DTL := #Data.StartTime[5], IN_SDTL := #Rmp5.Duration);
 #dint_elapsed := TIME_TO_DINT(#elapsed);
 #RM.Elapsed := "TimeINT_TO_SDTL"(#dint_elapsed);

 // Convert Time to Double Interager - Time Basic
 #elapsed := T_DIFF(IN1 := "General".CPUDTL, IN2 := #Data.StartTime[5]);
 #dint_elapsed := TIME_TO_DINT(#elapsed);
 #Rmp5.Elapsed := "TimeINT_TO_SDTL"(#dint_elapsed);

 // Set Flow SV
 #RM.FlowSV := #Rmp5.ValStart;
 #Rmp5.FlowSV := #Rmp5.ValStart;

 // Calculate Time Remaining
 #TimePV := T_DIFF(IN1 := #Data.FinishTime[5], IN2 := "General".CPUDTL);
 #TimePV_I := TIME_TO_DINT(#TimePV);
 #RM.CurrentStage := #RM.Seq;

 // Sequence Time Finished - Increment Sequence
 IF #TimePV_I <= 0 THEN
 #RM.FlowSV := #Rmp5.ValFinish;
 #Rmp5.FlowSV := #Rmp5.ValFinish;
 #RM.Seq := 0;
 #RM.CurrentStage := 0;
 #Rmp5.Follow := FALSE;
 END_IF;

 // Calculate Time Data - Reamining Time Fraction
 #TempDint := TIME_TO_DINT(T_DIFF(IN1 := #Data.FinishTime[5], IN2 := "General".CPUDTL));
 #TempDuration := TIME_TO_DINT(T_DIFF(IN1 := #Data.FinishTime[5], IN2 := #Data.StartTime[5]));
 #TempDint := REAL_TO_INT((DINT_TO_REAL(#TempDint) / DINT_TO_REAL(#TempDuration)) * 100.0);

 // Progress based on Remaining Time Fraction
 IF #TempDint > 0 THEN
 #Rmp5.Progress := 100 - DINT_TO_INT(#TempDint);
 END_IF;

END_IF;

// End Program

## RampMPC

RampMPC initiates the following commands:

1. Gets a Flow Rate Set Value and Start/Stop commands From “RampCtl”
2. Checks for previously store Operational Value for the same Setpoint – Applies the values if present
3. Calculates the Error between the Set Value and the actual Process Value
4. Starts in “Maxi” mode – driving just the Large Valve and continually monitoring the Flow Transmitter Feedback
5. Calculates the change in the Error (Delta) after Valve change
6. Predicts a future “Horizon” Process Value based on time and Delta data – Creates a Horizon based Error
7. Makes Large Valve changes as required to achieve the Flow Set Value.
8. If the Large Valve is trying to operate to it’s maximum or minimum capacity, the logic will move to “Mini” mode
9. In “Mini” mode the logic can use either just the small valve (low flow situations) or employ both valves (high flow situations)

// ***** Function Block - "RAMP_MPC" ***** //

// *** Ramp MPC - Valve Manipulation *** //
(*
Data Sequence "#Data.Seq"
 0: Flow Unstable - Adjusting
 1: Flow stable - Actual Error < 3 for 10 cycles
 2: Flow Stable/Store Operation Code - Actual Error < 1 for 10 cycles

Store Data Sequence "##StrData.Sequence"
 0:
 1: Stored Data = Operation Data
 2:
 3: Step to Sequence 4
 4:
 5:
 6:
 7: Valve Position Data Recorded - #Data.Seq = 2

WinCC Data Sequence "#WinCCData.Seq"
 0: Off


*)
// Increment Data Counter
"INC"(#Data.count);

// Set intial values
#Data.pv_ave := #WinCCData.FlowPV;
#qual := false;
#StoreRdy := false;
#real_FlowSV := (ROUND_REAL(#WinCCData.FlowSV * 100.0));
#Op_code := REAL_TO_DWORD(#real_FlowSV);

// Check Operation Code, Reset Sequence & Retreive index
IF #Op_code <> #StrData.OperationCode THEN
 #StrData.OperationCode := #Op_code;
 #StrData.Sequence := 1;
 #Data.Seq := 0;
 #Data.Gain_stored := false;
 #WinCCData."#Stored" := #StrData.IndexEnd;
END_IF;

// Set Process Value Delta
IF #Data.count > 10 THEN
 #Data.deltaPV:= #Data.pv_ave - #Data.lastPV;
 #Data.lastPV := #Data.pv_ave;
 #Data.count := 0;
END_IF;

// WinCC Sequence 0 (OFF) - Close Valves, Reset ReEstablish counter & Jump to MV Output
IF #WinCCData.Seq = 0 OR #WinCCData.FlowSV=0 THEN
 #Data.MV_SM := 0;
 #Data.MV_LG := 0;
 #WinCCData.FlowSV := 0;
 #Data.ReEstablishSec := 30;
 #StrData.OperationCode := 0;
 GOTO ACTUATE;
END_IF;

// Move Valve Feedback Data to WinCC
#WinCCData.VLgePV := #VLV_LG.POS_PV;
#WinCCData.VSmlPV := #VLV_SM.POS_PV;

// Convert Flow Set Value to an Interager
#int_FlowSV := REAL_TO_INT(#WinCCData.FlowSV );

// Sequence 3 - Step to Sequence 4 & Reset ReEstablish counter
IF #StrData.Sequence = 3 THEN
 #StrData.Sequence := 4;
 #Data.ReEstablishSec := 30;
END_IF;

// Sequence 5 - Copy Stored Valve Postion Data & Jump to MV Output
IF #StrData.Sequence = 5 THEN
 #Data.MV_SM := #StrData.Ret_POS_SML;
 #Data.MV_LG := #StrData.Ret_POS_LG;
 IF #Data.ReEstablishSec > 0 THEN
 "DEC"(#Data.ReEstablishSec);
 GOTO ACTUATE;
 ELSE
 #StrData.Sequence := 0;
 #Data.Mode := #Mini; // Set Data Mode
 END_IF;
END_IF;

// Sequence 6 - Read Failure
IF #StrData.Sequence = 6 THEN
 #Data.ReEstablishSec := 0;
 #StrData.Sequence := 0;
END_IF;

// Set Data Mode - Mini
IF #WinCCData.FlowSV <= 12 THEN
 #Data.Mode := #Mini;
END_IF;

// Read Stored Valuve Data
IF #Data.ReEstablishSec > 0 THEN
 // Check Flow Set Value Parameters
 IF "IN_RANGE_int"(MAX := 999, MIN := 2, VALUE := #int_FlowSV) THEN
 // Adjust Flow Set Value
 IF #StrVPos[#int_FlowSV] > 0 THEN
 #tempI:= #StrVPos[#int_FlowSV];
 END_IF;
 IF #tempI = 0 AND #StrVPos[#int_FlowSV - 1] <> 0 THEN
 #tempI := #StrVPos[#int_FlowSV - 1];
 END_IF;
 IF #tempI = 0 AND #StrVPos[#int_FlowSV + 1] <> 0 THEN
 #tempI := #StrVPos[#int_FlowSV + 1];
 END_IF;
 END_IF;
 // Decrement ReEstablish Counter
 "DEC"(#Data.ReEstablishSec);
 // Write Large Valve MV
 IF #StrData.Sequence <> 5 THEN
 IF #int_FlowSV <> 0 THEN
 #Data.MV_LG := #tempI;
 END_IF;
 // Write Small Valve MV
 #Data.MV_SM := 20;
 END_IF;
 // Reset Sequence after count down
 IF #Data.ReEstablishSec = 0 THEN
 #StrData.Sequence := 0;
 END_IF;
END_IF;

// ReEstablish Counter > 0
IF #Data.ReEstablishSec > 0 THEN
 GOTO ACTUATE;
END_IF;

// Calculate Horizon Process Value
#Data.HOZ_PV := #WinCCData.FlowPV + #Data.deltaPV;

// Calculate Horizon Error
#ERROR_FLOW := #WinCCData.FlowSV - #Data.HOZ_PV;

// Calculate Actual Error
#REALERROR_FLOW := #WinCCData.FlowSV - #WinCCData.FlowPV;

// Re-Calculte Horizon Process Value
#Data.HOZ_PV := #WinCCData.FlowPV + (#Data.deltaPV * #Data.HOZ_Time);

// Calculate Gain Data Index - Large Valve
IF ABS(#REALERROR_FLOW) <3 THEN
 IF #Data.Flow_count < 10 THEN
 "INC"(#Data.Flow_count);
 IF "IN_RANGE_int"(MAX :=100, MIN := 0, VALUE := #WinCCData.VLgeSV) THEN
 #Data.ValveGain[#WinCCData.VLgeSV] := #WinCCData.FlowPV / #WinCCData.VLgeSV;
 END_IF;
 #Data.Seq := 0;
 ELSE
 // Flow Stable
 #Data.stable := true;
 #Data.Seq:= 1;
 IF "IN_RANGE_int"(MAX :=1000, MIN := 0, VALUE := #int_FlowSV) THEN
 // Store Valve Position - Large Valve
 #StrVPos[#int_FlowSV] := REAL_TO_INT(#Data.MV_LG);
 #Data.ReEstablishSec := 0;
 END_IF;
 #Data.Mode := #Mini;
 #Data.Flow_count := 100;
 END_IF;
ELSE
 #Data.Seq := 0;
 IF #Data.Flow_count > 0 THEN
 "DEC"(#Data.Flow_count);
 ELSE
 // Flow NOT Stable
 #Data.stable := false;
 #StoreRdy := false;
 #Data.Mode := #Maxi;
 END_IF;
END_IF;

// Data Mode - Maxi - Large Valve
IF #Data.Mode = #Maxi THEN
 // Set Horizon Time
 #Data.HOZ_Time := 30;
 // Calculate Gain Data
 IF #Data.ValveGain[#WinCCData.VLgeSV] > 0 AND #WinCCData.VLgeSV>0 THEN
 #Data.Gain := #Data.ValveGain[#WinCCData.VLgeSV] * #Data.HOZ_Time;
 ELSE
 #Data.Gain := 6;
 END_IF;
 // Calculate Volume
 #Volumne := #REALERROR_FLOW / #Data.Gain;
 // Calculate Lage Valve MV
 #Data.MV_LG := #Data.MV_LG + #Volumne;
 // Set Data Mode
 IF #Data.MV_LG >= 100 OR #Data.MV_LG <=0 THEN
 #Data.Mode := #Mini;
 END_IF;
END_IF;

// Data Mode - Mini - Large & Small Valves
IF #Data.Mode = #Mini THEN
 #Data.HOZ_Time := 15;
 #int_FlowSV := #WinCCData.VLgePV;
 #Data.Gain := 5;
 #Volumne := #REALERROR_FLOW / #Data.Gain;
 #Data.MV_SM := #Data.MV_SM + #Volumne;

 // Adjust Large & Small Valve MV
 IF #Data.MV_LG < 100 THEN
 IF #Data.MV_SM >= 100 THEN
 #Data.MV_LG := #Data.MV_LG + 1;
 #Data.MV_SM := #Data.MV_SM - 20;
 END_IF;
 END_IF;

 IF #Data.MV_SM <= 0 THEN
 #Data.MV_LG := #Data.MV_LG - 1;
 #Data.MV_SM := #Data.MV_SM + 20;
 END_IF;
 // Check Actual Flow Error
 IF ABS(#REALERROR_FLOW) < 1 THEN
 IF #Data.Flow_count < 10 THEN
 "INC"(#Data.Flow_count);
 ELSE
 #Data.Seq := 2;
 END_IF;
 ELSE
 #Data.Flow_count := 0;
 #Data.Seq := 1;
 END_IF;
END_IF;

// Sequence 2 - Store Valve Data
IF #Data.Seq = 2 THEN
 // Operation Code (Line 15) -- WinCC SV * 100
 #StrData.OperationCode := #Op_code;
 IF #Data.Gain_stored = false THEN
 "INC"(#WinCCData."#Stored");
 #Data.Gain_stored := true;
 END_IF;
 #StrData.Str_POS_LG := REAL_TO_INT(#Data.MV_LG);
 #StrData.Str_POS_SML := REAL_TO_INT(#Data.MV_SM);
 #StrData.Sequence := 7;
 #Data.Seq := 0;
 #Data.Flow_count := 0;
END_IF;

// Actuate - Check & Output Manipulated Variable
ACTUATE:
// Check MV Parameters
IF #Data.MV_SM > 100 THEN
 #Data.MV_SM := 100;
END_IF;
IF #Data.MV_SM < 0 THEN
 #Data.MV_SM := 0;
END_IF;
IF #Data.MV_LG > 100 THEN
 #Data.MV_LG := 100;
END_IF;
IF #Data.MV_LG < 0 THEN
 #Data.MV_LG := 0;
END_IF;

 // Small Valve - Auto
IF #VLV_SM.STS = 2 THEN
 #WinCCData.VSmlSV := REAL_TO_INT(ABS(#Data.MV_SM));
 #VLV_SM.POS_SV := REAL_TO_INT(ABS(#Data.MV_SM));
 END_IF;
 // Large Vlave - Auto
IF #VLV_LG.STS = 2 THEN
 #WinCCData.VLgeSV := REAL_TO_INT(ABS(#Data.MV_LG));
 #VLV_LG.POS_SV := REAL_TO_INT(ABS(#Data.MV_LG));
END_IF;
