# Supplementary material for: Automated flow control of a multi-lane swimming chamber for small fishes indicates species-specific sensitivity to experimental protocols
Source: Conserv Physiol. 2021 Jan 7;9(1):coaa131. doi: 10.1093/conphys/coaa131 (PMC7905161; doi:10.1093/conphys/coaa131)
Supplement: Supp_material_S3_coaa131 [file supp_material_s3_coaa131.docx]

**Supplementary material S3**

Automated flow control of a multi-lane swimming chamber for small fishes indicates species-specific sensitivity to experimental protocols
**Illing et al 2020 Conservation Physiology**

Legend:
Ucrit_cm_s = Critical swimming speed (*U*_crit_, cm s^-1^)
SL_mm_mean = Standard length (mm)
Interval_min = Step length during *U*_crit_ protocol (min)
Increment_cm_s = Step height during *U*_crit_ protocol (cm s^-1^)

**GAM model parameters for Fig. 5A (*Amphiprion melanopus*)**

Family: gaussian
Link function: identity

Formula:
Ucrit_cm_s ~ s(SL_mm_mean) + Interval_min + Increment_cm_s

Parametric coefficients:
 Estimate Std. Error t value Pr(>|t|)
(Intercept) 16.2361 0.3868 41.980 < 2e-16 ***
Interval_min5 -0.4876 0.4468 -1.091 0.276272
Interval_min10 -1.5795 0.4521 -3.494 0.000574 ***
Interval_min20 -2.7263 0.4466 -6.104 4.53e-09 ***
Increment_cm_s1.5 0.1830 0.3869 0.473 0.636661
Increment_cm_s2 0.7226 0.3949 1.830 0.068600 .
---
Signif. codes: 0 '***' 0.001 '**' 0.01 '*' 0.05 '.' 0.1 ' ' 1

Approximate significance of smooth terms:
 edf Ref.df F p-value
s(SL_mm_mean) 6.849 7.969 221.8 <2e-16 ***
---
Signif. codes: 0 '***' 0.001 '**' 0.01 '*' 0.05 '.' 0.1 ' ' 1

R-sq.(adj) = 0.885 Deviance explained = 89.1%
-REML = 551.52 Scale est. = 5.8814 n = 236

Post-hoc comparisons for intervals (using the ‘glht’ package):

Linear Hypotheses:
 Estimate Std. Error z value Pr(>|z|)
2 - 5 == 0 -0.4876 0.4468 -1.091 0.69479
2 - 10 == 0 -1.5795 0.4521 -3.494 0.00263 **
2 - 20 == 0 -2.7263 0.4466 -6.104 < 0.001 ***
5-10 == 0 -1.0919 0.4538 -2.406 0.07595 .
5-20 == 0 -2.2386 0.4473 -5.004 < 0.001 ***
10-20 == 0 -1.1467 0.4534 -2.529 0.05579 .
---
Signif. codes: 0 '***' 0.001 '**' 0.01 '*' 0.05 '.' 0.1 ' ' 1
(Adjusted p values reported -- single-step method)

Post-hoc comparisons for increments (using the ‘glht’ package):

Linear Hypotheses:
 Estimate Std. Error z value Pr(>|z|)
1 - 1.5 == 0 0.1830 0.3869 0.473 0.884
1 - 2 == 0 0.7226 0.3949 1.830 0.160
1.5 - 2 == 0 0.5396 0.3915 1.378 0.352
(Adjusted p values reported -- single-step method)

**Linear model parameters for Fig. 5B (*Lates calcarifer*)**

lm(formula = Ucrit_cm_s ~ SL_mm_mean * Interval_min * Increment_cm_s,
 data = LC_fishdata_SW_M_mod)

Residuals:
 Min 1Q Median 3Q Max
-5.8911 -0.9180 -0.0887 0.7720 7.2383

Coefficients:
 Estimate Std. Error t value Pr(>|t|)
(Intercept) -4.99207 1.88303 -2.651 0.00930 **
SL_mm_mean 2.00688 0.30357 6.611 1.78e-09 ***
Interval_min5 -3.27746 2.77534 -1.181 0.24038
Interval_min10 -0.36656 2.77796 -0.132 0.89528
Increment_cm_s0.5 7.63686 2.85067 2.679 0.00861 **
Increment_cm_s1 -0.15265 3.14543 -0.049 0.96139
SL_mm_mean:Interval_min5 0.38914 0.46410 0.838 0.40372
SL_mm_mean:Interval_min10 0.19988 0.46348 0.431 0.66719
SL_mm_mean:Increment_cm_s0.5 -1.52838 0.46873 -3.261 0.00151 **
SL_mm_mean:Increment_cm_s1 0.01306 0.54603 0.024 0.98096
Interval_min5:Increment_cm_s0.5 1.88310 4.18711 0.450 0.65385
Interval_min10:Increment_cm_s0.5 -6.44627 4.04238 -1.595 0.11388
Interval_min5:Increment_cm_s1 9.29502 4.14779 2.241 0.02720 *
Interval_min10:Increment_cm_s1 4.83374 4.43749 1.089 0.27859
SL_mm_mean:Interval_min5:Increment_cm_s0.5 -0.23496 0.69797 -0.337 0.73708
SL_mm_mean:Interval_min10:Increment_cm_s0.5 0.82994 0.67229 1.234 0.21986
SL_mm_mean:Interval_min5:Increment_cm_s1 -1.43862 0.70662 -2.036 0.04435 *
SL_mm_mean:Interval_min10:Increment_cm_s1 -1.19487 0.75339 -1.586 0.11584
---
Signif. codes: 0 '***' 0.001 '**' 0.01 '*' 0.05 '.' 0.1 ' ' 1

Residual standard error: 2.177 on 102 degrees of freedom
 (15 observations deleted due to missingness)
 Multiple R-squared: 0.6762, Adjusted R-squared: 0.6222
 F-statistic: 12.53 on 17 and 102 DF, p-value: < 2.2e-16

Post-hoc comparisons (using the ‘emmeans’ package):

emmeans(mod.lm4, specs = pairwise ~ Interval_min|Increment_cm_s, type = "response")

$emmeans
Increment_cm_s = 0.25:
 Interval_min emmean SE df lower.CL upper.CL
 2 6.55 0.564 102 5.43 7.67
 5 5.51 0.565 102 4.39 6.64
 10 7.34 0.585 102 6.18 8.50

Increment_cm_s = 0.5:
 Interval_min emmean SE df lower.CL upper.CL
 2 5.40 0.629 102 4.15 6.64
 5 4.89 0.583 102 3.73 6.05
 10 4.51 0.582 102 3.35 5.66

Increment_cm_s = 1:
 Interval_min emmean SE df lower.CL upper.CL
 2 6.48 0.651 102 5.18 7.77
 5 6.46 0.607 102 5.25 7.66
 10 5.22 0.661 102 3.91 6.53

Confidence level used: 0.95

$contrasts
Increment_cm_s = 0.25:
 contrast estimate SE df t.ratio p.value
 2 - 5 1.0388 0.799 102 1.300 0.3983
 2 - 10 -0.7833 0.813 102 -0.964 0.6013
 5 - 10 -1.8221 0.813 102 -2.240 0.0693

Increment_cm_s = 0.5:
 contrast estimate SE df t.ratio p.value
 2 - 5 0.5074 0.857 102 0.592 0.8247
 2 - 10 0.8885 0.857 102 1.037 0.5554
 5 - 10 0.3811 0.824 102 0.463 0.8889

Increment_cm_s = 1:
 contrast estimate SE df t.ratio p.value
 2 - 5 0.0199 0.891 102 0.022 0.9997
 2 - 10 1.2568 0.928 102 1.355 0.3686
 5 - 10 1.2369 0.897 102 1.378 0.3560

P value adjustment: tukey method for comparing a family of 3 estimates

**Linear model parameters for Fig. 6A (*Amphiprion melanopus*)**

lm(formula = log(Grid_time_min) ~ Interval_min + Increment_cm_s +
 SL_mm_mean, data = AM_grid_time)

Residuals:
 Min 1Q Median 3Q Max
-3.7578 -0.4838 0.1108 0.7265 1.9788

Coefficients:
 Estimate Std. Error t value Pr(>|t|)
(Intercept) 2.07817 0.36726 5.659 6.13e-08 ***
Interval_min5 1.10237 0.22370 4.928 1.92e-06 ***
Interval_min10 1.40145 0.23189 6.044 8.85e-09 ***
Interval_min20 1.60818 0.22373 7.188 1.83e-11 ***
Increment_cm_s1.5 -0.50220 0.19478 -2.578 0.0108 *
Increment_cm_s2 -0.92740 0.19964 -4.645 6.65e-06 ***
SL_mm_mean -0.06355 0.05040 -1.261 0.2090
---
Signif. codes: 0 '***' 0.001 '**' 0.01 '*' 0.05 '.' 0.1 ' ' 1

Residual standard error: 1.084 on 175 degrees of freedom
Multiple R-squared: 0.3257, Adjusted R-squared: 0.3025
F-statistic: 14.09 on 6 and 175 DF, p-value: 4.682e-13

Post-hoc comparisons (using the ‘emmeans’ package):

emmeans(AM_grid_lm3,~Interval_min|Increment_cm_s,type="response")

Increment_cm_s = 1:
 Interval_min response SE df t.ratio p.value
 2 5.35 1.039 175 8.644 <.0001
 5 16.12 3.164 175 14.166 <.0001
 10 21.74 4.370 175 15.320 <.0001
 20 26.74 5.245 175 16.750 <.0001

Increment_cm_s = 1.5:
 Interval_min response SE df t.ratio p.value
 2 3.24 0.627 175 6.073 <.0001
 5 9.76 1.889 175 11.765 <.0001
 10 13.16 2.639 175 12.849 <.0001
 20 16.18 3.132 175 14.384 <.0001

Increment_cm_s = 2:
 Interval_min response SE df t.ratio p.value
 2 2.12 0.418 175 3.804 0.0002
 5 6.38 1.244 175 9.498 <.0001
 10 8.60 1.820 175 10.169 <.0001
 20 10.58 2.065 175 12.079 <.0001

Tests are performed on the log scale

**Linear model parameters for Fig. 6B (*Lates calcarifer*)**

lm(formula = log(Grid_time_min) ~ Interval_min * Increment_cm_s +
 SL_mm_mean, data = LC_grid_time)

Residuals:
 Min 1Q Median 3Q Max
-2.9040 -0.6044 0.1791 0.7029 2.0845

Coefficients:
 Estimate Std. Error t value Pr(>|t|)
(Intercept) 1.7499 0.4914 3.561 0.000615 ***
Interval_min5 1.3018 0.4391 2.965 0.003950 **
Interval_min10 1.8524 0.4490 4.125 8.75e-05 ***
Increment_cm_s0.5 -0.6538 0.4737 -1.380 0.171229
Increment_cm_s1 -1.0227 0.4759 -2.149 0.034546 *
SL_mm_mean 0.1517 0.0660 2.299 0.024011 *
Interval_min5:Increment_cm_s0.5 -0.4373 0.6535 -0.669 0.505242
Interval_min10:Increment_cm_s0.5 -0.7074 0.6596 -1.072 0.286616
Interval_min5:Increment_cm_s1 -0.9694 0.6643 -1.459 0.148224
Interval_min10:Increment_cm_s1 0.2612 0.6944 0.376 0.707705
---
Signif. codes: 0 '***' 0.001 '**' 0.01 '*' 0.05 '.' 0.1 ' ' 1

Residual standard error: 1.074 on 83 degrees of freedom
Multiple R-squared: 0.4709, Adjusted R-squared: 0.4136
F-statistic: 8.209 on 9 and 83 DF, p-value: 1.258e-08

Post-hoc comparisons (using the ‘emmeans’ package):

emmeans(LC_grid_lm4,~Increment_cm_s|Interval_min,type="response")

Interval_min = 2:
 Increment_cm_s response SE df t.ratio p.value
 0.25 13.57 4.21 83 8.407 <.0001
 0.5 7.05 2.53 83 5.457 <.0001
 1 4.88 1.76 83 4.402 <.0001

Interval_min = 5:
 Increment_cm_s response SE df t.ratio p.value
 0.25 49.87 15.48 83 12.596 <.0001
 0.5 16.75 5.43 83 8.690 <.0001
 1 6.80 2.31 83 5.641 <.0001

Interval_min = 10:
 Increment_cm_s response SE df t.ratio p.value
 0.25 86.48 28.04 83 13.753 <.0001
 0.5 22.17 7.18 83 9.568 <.0001
 1 40.39 15.39 83 9.708 <.0001

Tests are performed on the log scale
